# Association of health behaviors with healthcare workers’ physical and psychological well-being: Learning from the COVID-19 pandemic

**DOI:** 10.1371/journal.pone.0334752

**Published:** 2025-10-31

**Authors:** Elie Mulhem, Lori Lackman Zeman, Karen Childers, Sujoy Roy, Ramin Homayouni

**Affiliations:** 1 Department of Family Medicine, Corewell Health Beaumont Troy, Troy, Michigan, United States of America; 2 Department of Family Medicine and Community Health, Oakland University William Beaumont School of Medicine, Rochester, Michigan, United States of America; 3 The Department of Public Health Sciences, Division of Biostatistics, Henry Ford Health, Detroit, Michigan, United States of America; 4 Department of Foundational Medical Studies, Oakland University William Beaumont School of Medicine, Rochester, Michigan, United States of America; National Institutes of Health, University of the Philippines Manila / De La Salle University, PHILIPPINES

## Abstract

**Background:**

During the COVID-19 pandemic healthcare workers reported using a variety of positive or negative health behaviors as a strategy to cope with the increased stress at work and home. This study examined the extent of healthcare workers’ engagement in various health behaviors and the association of these behaviors with changes in self-reported physical and psychological well-being during the pandemic.

**Methods:**

A survey was developed and administered to healthcare workers in a large healthcare system in Southeast Michigan between November 2, 2020, and January 21, 2021, during Michigan’s second COVID-19 surge.

**Results:**

During the study period 368 healthcare workers completed the survey. The majority of participants were female (83.2%) and aged between 25−34 years (29.3%). Most participants reported a drop in their psychological well-being and physical health during the pandemic. Overall, 14.8% to 47.7% of participants rated their engagement in a positive health behavior as “often” or “very often”. Engagement in most positive health behaviors was associated with higher self-rating of physical health and psychological well-being. The estimated decrease in psychological well-being scores for participants who often/very often *engaged in pleasurable activities* was 0.91 points less (p = 0.003, 95% CI [−1.50, −0.32]) than for those who rarely/never engaged in such activity. *Exercise*, *healthy eating*, *getting enough sleep*, *engaging in pleasurable activities*, *focusing on gratitude and positive things*, and *getting social support* appeared to positively influence the degree to which participant’s physical health declined. After controlling for age, gender and other potential confounders, each additional positive health behavior was associated with a 0.22-point decrease in the rate of decline in overall physical health (p = 0.000, 95% CI −0.35, −0.10).

**Discussion:**

Healthcare workers who regularly engaged in positive health behaviors during the pandemic reported better psychological and physically well-being than those who did not before and during COVID-19. *Engaging in pleasurable activities* was the only health behavior in our study that demonstrated a potential protective effect against the decline in psychological well-being during the pandemic. Several other positive health behaviors were associated with a lower rate of decline of physical health during the pandemic. Health systems should consider implementing strategies to increase opportunities for pleasurable activities for healthcare workers, both in the workplace and at home, especially during times of increased stress.

## Introduction

The COVID-19 pandemic had a significant emotional and physical impact on the entire population, especially on healthcare workers [1–4]. During periods of stress, individuals often adopt various health behaviors to help them cope and manage stress [5]. A health behavior is defined by the Center for Disease Control and Prevention as: “action taken by an individual or group to change or maintain their health status or prevent illness or injury”. Changes in health behaviors in response to stress can include negative behaviors such as increased alcohol consumption, smoking and unhealthy eating, as well as positive behaviors, such as increased exercise and prayer [5,6].

During the COVID-19 pandemic, healthcare workers endorsed using a variety of health behaviors to cope with the increased stress at work and at home, including seeking support and communication with family and friends, exercising, prayer, music, yoga, meditation, journaling, and writing in a gratitude journal [7–10].

In this study we examined the extent to which healthcare workers used various health behaviors during the COVID-19 pandemic and the association of these behaviors with changes in self-reported physical health status and psychological well-being during the pandemic. We hypothesized that positive health behaviors would protect against declines in physical and psychological wellbeing of healthcare workers during the high stress period of the COVID-19 pandemic, while negative health behaviors would be associated with a steeper rate of decline.

## Methods

### Setting and study population

This study took place in a large healthcare system in Southeast Michigan, consisting of eight hospitals with 3,375 beds and 155 outpatient sites. Data reported here was collected as part of a larger qualitative study in which healthcare workers were asked to record up to 5 minutes of reflection on their experiences during the pandemic at two different time points [11,12] Time point 1 (April to June 2020) corresponding to the initial COVID-19 surge in Southeast Michigan, as measured by the number of patients hospitalized with COVID-19 across the study healthcare system (S1 Fig). Time point 2 (November 2, 2020, and January 21, 2021) corresponding to the second COVID-19 surge. During the second time point, a written survey was added to gather quantitative, descriptive information regarding the impact COVID-19 was having on specific domains and to understand health behaviors and coping strategies that healthcare workers were utilizing during this high stress period. Recruitment targeted all healthcare workers, not only those on the frontline or involved in direct patient care. Invitations to participate were periodically included in daily COVID-19 update emails sent out by the health system’s communication department to all employees and contracted workers. The study was approved by the health system’s Institutional Review Board, and a Waiver of Consent Documentation was granted. Participants were required to read an information sheet describing the study, on the study website landing page and indicate understanding of its content by clicking “I Acknowledge” button before participating in the study.

### Data collection

The recruitment invitation directed interested participants to a dedicated website (COVID19HERO.health), where they could consent to participate, provide basic demographic information, and then had the option to answer survey questions, submit an audio recording (data from audio recording reported separately) or do both [11,12]. Participants were instructed not to include any personal or patient identifiers in their survey responses.

### Survey

The survey was developed specifically for this study to capture quantitative, descriptive information on work life, personal life, emotions and health, and coping strategies. It was informed by what we learned from the qualitative data we collected during time Point 1 of the study, our own clinical observations, and professional recommendations for coping strategies during COVID-19. [13] Participants were presented with a list of items relevant to work life, personal life, and emotions and health and were asked to rate how these compared to life prior to the COVID-19 pandemic (significantly less, less, about the same, more, significantly more). Additionally, participants provided a global rating of their psychological well-being and physical health both before and during the pandemic on a 1–10 Likert scale (1 = very poor, to 10 = very good). Finally, participants were asked to rate how frequently they engaged in various health behaviors and coping strategies during COVID-19, measured on a 1–5 Likert scale (1 = never, 2 = rarely, 3 = sometimes, 4 = often, 5 = very often). Health behaviors and coping strategies were selected to reflect those commonly recommended by healthcare professionals, and the World Health Organization during COVID-19 [13]. See S2 Fig for a copy of the survey.

### Statistical analysis

The primary aims of the analyses were to: (1) compare physical health and psychological well-being ratings during the pandemic between patients who engaged in health behaviors often or very often versus rarely or never, (2) explore the change in psychological well-being and physical health scores from before to during the pandemic, (3) identify participant behaviors associated with the change in these ratings, and(4) examine the association between the number of health behaviors a participant engages in and the change in physical health and psychological well-being ratings. A *p*-value < 0.05 was considered statistically significant.

#### Survey completion bias.

Before conducting analyses for the primary aims, potential survey completion bias was explored. Some participants consented to the study and provided demographic and job-related information but did not take the survey. Characteristics of all participants were summarized using frequency and percentage and compared between those who completed the survey and those who did not complete the survey using Fisher’s exact tests.

#### Change in physical health and psychological well-being.

Psychological well-being and physical health ratings were combined into 3 categories [1–10] due to low counts. The shift in scores from before to during the pandemic were visualized using alluvial diagrams. A McNemar-Bowker test was used to determine if there was a significant shift in the distribution of scores from before to during the pandemic. For each participant the change in psychological well-being and physical health rating was calculated by subtracting the pre-pandemic ratings from the ratings during the pandemic. As such, a negative value indicates a decrease in self-reported psychological well-being and physical health from before to during the pandemic.

#### Frequency of engagement in health behaviors.

The frequency of reported engagement in positive (*exercise, healthy eating, getting enough sleep, mindfulness or relaxation exercise, focusing on gratitude and positive things, engaging in pleasurable activities, and getting social support*) and negative (*tobacco, alcohol and illicit drug use*) health behaviors during the pandemic were collapsed into 3 groups (rarely/never, sometimes, often/very often) for ease of interpretation. For each health behavior, the percent of participants in each frequency group were calculated and displayed graphically. Wilcoxon rank-sum tests were conducted to compare physical health and psychological well-being during the pandemic between participants who engaged in the health behavior often/very often vs rarely/never.

#### Association between change in physical health and psychological well-being and health behaviors.

Separate adjusted linear models were used to explore the association between each health behavior and the change in psychological well-being or physical health ratings from before to during the pandemic. The dependent variable in each model was the change in psychological well-being or physical health rating with the health behavior frequency as an independent variable and the following factors: age, gender, worked in patient care, had school aged children, near high-risk relatives, and in high-risk group. The models were used to estimate the mean change in score between participants who engaged in the health behaviors often/very often versus rarely/never and to test the significance of this change. Residual versus fits plots and quantile-quantile plots for all linear models were inspected to verify the model assumptions were reasonable. All analyses were performed using R Statistical Software (v4.2.2; R Core Team 2022).

#### Association between change in physical health and psychological well-being and the number of health behaviors.

The frequency of engagement in positive health behaviors was then quantified by summing the number of positive health behaviors each respondent engaged in often or very often. The difference in physical health and psychological well-being ratings were stratified by different sums of healthy behaviors [3–5]. Multivariable linear models were used to evaluate the change in physical health and psychological well-being ratings from before to during the pandemic with difference in rating as the dependent variable and sum of healthy behaviors along with covariates of age, gender, worked in patients care, had school aged children, near high-risk relatives, and in high-risk group as independent variables.

## Results

During the study period, 508 healthcare workers accessed the website, and 368 completed the survey. There were no statistically significant differences in participant characteristics between individuals who completed the survey and those who did not (Table 1). The majority of survey participants were female (83.2%), 29.3% were between the ages of 25–34 years, and 68.5% were involved in direct patient care. In addition, the largest group of the survey participants were nurses (38.6%) or worked in a hospital inpatient setting (62.8%).

**Table 1:** Participant demographics. * P-values were determined using Fisher-Freeman-Halton test when contingency tables are larger than 2 × 2.

	All Participants	Did not Complete Survey	Completed Survey	P-value*
	N = 508 n (%)	N = 140 n (%)	N = 368 n (%)	
Age				0.47
18 - 24	34 (6.7)	14 (10.0)	20 (5.4)	
25 - 34	150 (29.5)	42 (30.0)	108 (29.3)	
35 - 44	114 (22.4)	30 (21.4)	84 (22.8)	
45 - 54	101 (19.9)	27 (19.3)	74 (20.1)	
55 - 64	94 (18.5)	25 (17.9)	69 (18.8)	
65 - 74	15 (3.0)	2 (1.4)	13 (3.5)	
Gender				0.492
Female	425 (83.7)	119 (85.0)	306 (83.2)	
Male	81 (15.9)	20 (14.3)	61 (16.6)	
Other	2 (0.4)	1 (0.7)	1 (0.3)	
Direct patient Care	340 (66.9)	88 (62.9)	252 (68.5)	0.24
Role				0.27
Administrative support services	17 (3.3)	6 (4.3)	11 (3.0)	
Administrator Manager	49 (9.6)	10 (7.1)	39 (10.6)	
Clerical	40 (7.9)	15 (10.7)	25 (6.8)	
Clinical support services	28 (5.5)	8 (5.7)	20 (5.4)	
Medical assistant or nursing assistant	31 (6.1)	13 (9.3)	18 (4.9)	
Medical provider	47 (9.3)	11 (7.9)	36 (9.8)	
Nurse	185 (36.4)	43 (30.7)	142 (38.6)	
Other	45 (8.9)	14 (10.0)	31 (8.4)	
Other health care provider	66 (13.0)	20 (14.3)	46 (12.5)	
Setting				0.21
Administrative office	40 (7.9)	14 (10.0)	26 (7.1)	
Both inpatient and outpatient clinical	48 (9.4)	19 (13.6)	29 (7.9)	
Inpatient hospital	313 (61.6)	82 (58.6)	231 (62.8)	
Other	49 (9.6)	12 (8.6)	37 (10.1)	
Outpatient clinical office	58 (11.4)	13 (9.3)	45 (12.2)	
Have school aged children	203 (40.1)	62 (44.3)	141 (38.5)	0.26
Have related high-risk people around	204 (40.3)	60 (42.9)	144 (39.3)	0.40
In high-risk group	169 (33.4)	53 (37.9)	116 (31.7)	0.20
Positive for COVID-19	114 (22.5)	39 (27.9)	75 (20.5)	0.09
Family members are COVID-19 Positive	150 (29.6)	46 (32.9)	104 (28.4)	0.33
Have direct contact with patients	332 (65.5)	88 (62.9)	244 (66.5)	0.46
Working at location during COVID-19	140 (27.6)	33 (23.6)	107 (29.2)	0.22

Most participants reported a decrease in the quality of life, with 72.6% reporting increased isolation from immediate family and 79.8% reporting decreased connectedness with friends (S3 Fig). In addition, most participants reported increased work stress (83.5%), increased workload (69.4%), feeling less safe at work (71.1%), as well as increases in stress (91.8%), loneliness (59%), anxiety (87.6%), and exhaustion (80.3%) during the pandemic (S4 and S5 Figs).

### Change in psychological well-being and physical health during the pandemic compared to before the pandemic

**Fig 1:**
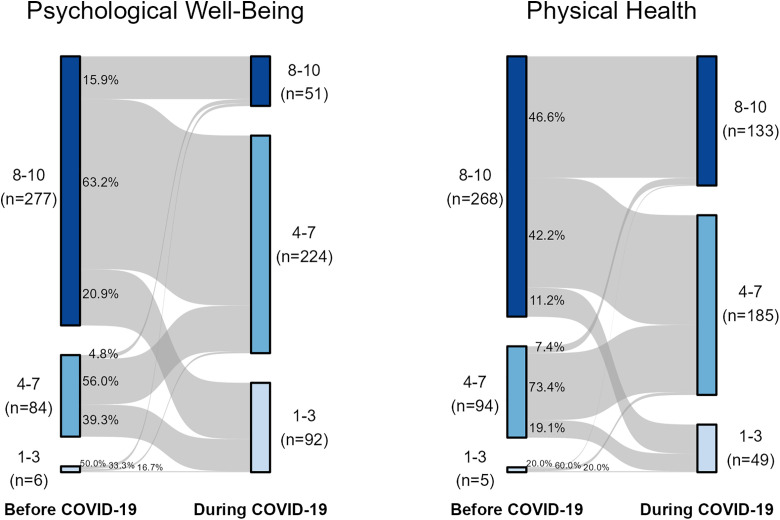
Self-rated psychological well-being and physical health of healthcare workers before and during the COVID-19 pandemic. Gray ribbons show how rating changed, with ribbon width representing the proportion of participants in each category.

We found a significant decrease in self-reported rating of psychological well-being (p < 0.001) and physical health (p < 0.001) during the COVID-19 pandemic compared to before the pandemic. On a scale of 1–10 (1 = very poor, 10 = very good), the majority of respondents (rated their pre-pandemic psychological well-being (75.4%) and physical health (73.0%) above 8 (Fig 1). Most participants reported a decline in both psychological well-being and physical health during the pandemic. Among participants who rated their pre-pandemic psychological well-being in the 8–10 range, only 15.9% reported the same rating during the pandemic, 62.3% dropped to 4–7 range, while 20.9% dropped to 1–3 range. With respect to physical health, among those who rated themselves 8–10 before the pandemic, 46.6% reported the same rating during the pandemic, 42.2% dropped to 4–7 range, and 11.2% dropped to 1–3 range (Fig 1).

### Health behaviors

**Fig 2:**
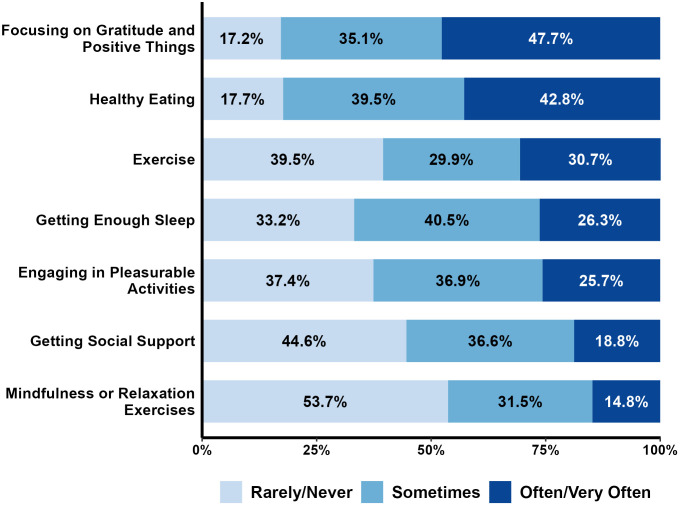
Distribution of participant responses for engaging in positive health behaviors during the pandemic.

**Fig 3:**
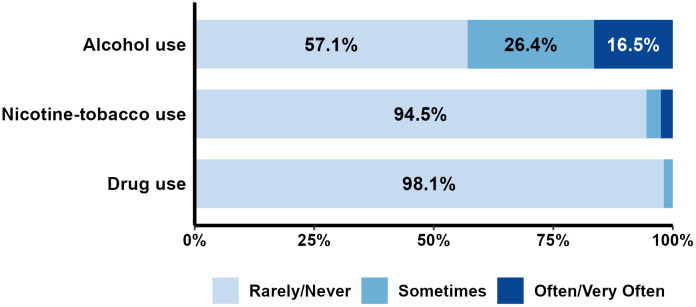
Distribution of participant responses for engaging in negative health behaviors during COVID-19. Percentages less than 5% are not labeled.

On a Likert scale of 1 (rarely) to 5 (very often), participants reported how often they engaged in seven common positive health behaviors and 3 negative health behaviors during the COVID-19 pandemic (Figs 2 and 3). The percentage of participants reporting a high level of engagement in positive health behaviors during the pandemic varied across behavior. Overall, 14.8% to 47.7% of participants rated their engagement in a particular behavior as “often” or “very often”. The positive health behaviors with the highest proportion of the participants who often or very often engaged in them were *focusing on gratitude and positive things* (47.7%) followed by *healthy eating* (42.8%). Conversely, a large proportion of participants rarely or never engaged in *mindfulness relaxation exercises* (53.7%) or *getting social support* (44.6%). Other health behaviors such as *exercise*, *getting enough sleep,* and *engaging in pleasurable activities* were more equally distributed across the scale.

Participants also reported using alcohol as a coping strategy during the pandemic, with 16.5% using alcohol often/very often and 26.4% using alcohol sometimes. Tobacco-use during the pandemic was reported by 8% of participants, while drug use was only reported by 1.9% of participants (Fig 3).

### Relationship between Health behaviors and psychological well-being and physical health

**Fig 4:**
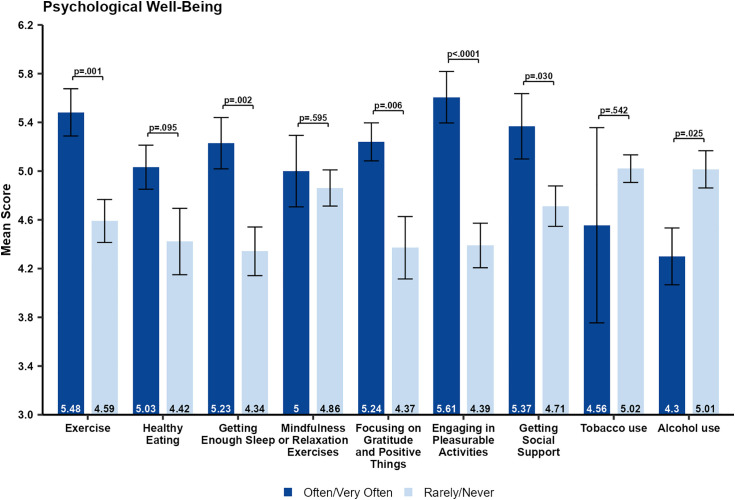
Comparison of self-reported psychological well-being scores during the COVID-19 pandemic by frequency of each health behavior. Data are shown as mean standard error and p-values are from Wilcoxon rank-sum tests.

**Fig 5:**
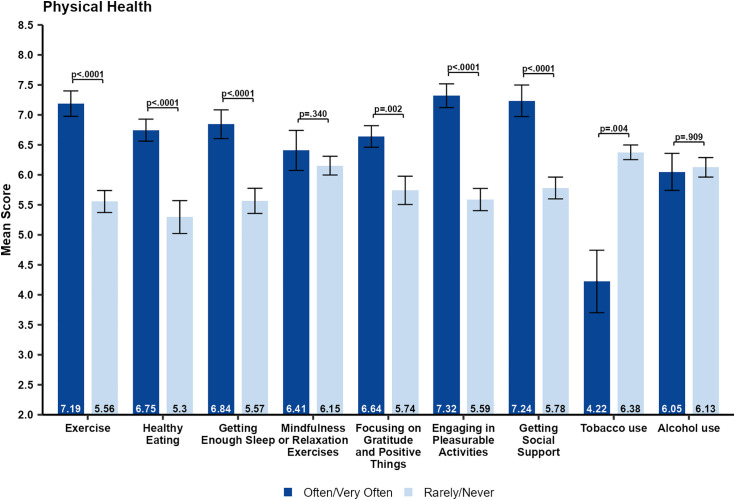
Comparison of self-reported physical health scores during the COVID-19 pandemic by frequency of health behaviors engagement. Data are shown as mean ± standard error and p-values are from Wilcoxon rank-sum tests.

As shown in Fig 1, most respondents reported a decrease in their psychological well-being and physical health during the pandemic. In general, the self-reported scores for psychological wellbeing (Fig 4) and physical health (Fig 5) during the pandemic were higher in healthcare workers who often/very often engaged in positive health behaviors. However, these individuals typically also reported higher baseline psychological well-being and physical health prior to the pandemic.

We compared those who reported often/very often to those who reported rarely/never engaged in different health behaviors during the pandemic with respect to psychological well-being (Fig 4) and physical health (Fig 5). Few participants reported any drug use, so it was not included in the analysis. Significantly higher self-reported psychological well-being during the pandemic was found for individuals who reported often/very often engaging in any of the following health behaviors: *exercise* (p = 0.001), *getting enough sleep* (p = 0.002), *focusing on gratitude and positive things* (p = 0.0006),), *getting social support* (p = 0.030), and *engaging in pleasurable activities* (p < 0.0001). While engaging in *healthy eating* (p = 0.95) and *mindfulness or relaxation exercises* (p = 0.595) were not associated with a significantly higher self-rating of psychological well-being. Participants who reported engaging in *alcohol use* often or very often had significantly lower self-reported psychological well-being compared to those who rarely or never engaged in such behaviors. For physical health, significantly higher self-rated physical health during the pandemic was found for individuals who often/very often reported engaging in any of the following health behaviors: *exercise* (p < 0.0001), *healthy eating* (P < 0.0001), *getting enough sleep* (p < 0.0001), *focusing on gratitude and positive things* (p = 0.002), *engaging in pleasurable activities* (p < 0.0001), and *getting social support* (p < 0.0001). Only *mindfulness or relaxation exercises* (p = 0.340) was not associated with a significantly higher self-rating of physical health. In addition, those who often/very often reported engaging in *tobacco use* had significantly (p = 0.004) lower self-rated physical health during the pandemic.

To evaluate if certain health behaviors were associated with the rate of decline in psychological well-being or physical health during the pandemic, we examined the changes in psychological well-being (Table 2) or physical health (Table 3) between individuals who reported they often/very often engaged in each health behavior compared to those who reported rarely/never engaged in the same health behavior. Adjustment was made for the possible confounders of age, gender, work in patient care, had school aged children, near high-risk relatives, and in high-risk group. Only two health behaviors were associated with the decline of psychological well-being during the pandemic. The estimated decrease in psychological well-being scores for respondents who often/very often *engaged in pleasurable activities* was 0.91 points less (p = 0.003, 95% CI [−1.50, −0.32]) than the estimated decrease for respondents who rarely/never engaged in this activity (Table 2). While the estimated decrease in psychological well-being scores was 1.04 points more (p = 0.002, 95% CI [0.39, 1.7] in participants who reported often/very often using alcohol compared to those who rarely/never reported using alcohol.

**Table 2:** Association of each health behavior and the reported decrease in psychological well-being during the pandemic between individuals who often/very often compared to rarely/never engaged in health behaviors. Results are predicted means and 95% confidence intervals from linear model analysis adjusted for age, gender, worked in patient care, had school aged children, near high-risk relatives and in high-risk group. Negative values indicate a reduction in psychological well-being decline while positive values indicate an increase in physical health decline.

Health Behavior	Estimate (95% CI)	p-value
Exercise	−0.12 (−0.69, 0.46)	0.692
Healthy Eating	−0.11 (−0.78, 0.57)	0.756
Getting Enough Sleep	−0.41 (−1.01, 0.19)	0.178
Mindfulness or Relaxation Exercises	−0.16 (−0.85, 0.54)	0.652
Focusing on Gratitude and Positive Things	−0.27 (−0.95, 0.41)	0.432
Engaging in Pleasurable Activities	**−0.91 (−1.5, −0.31)**	**0.003**
Getting Social Support	−0.54 (−1.19, 0.12)	0.107
Tobacco Use	−0.04 (−1.56, 1.47)	0.956
Alcohol Use	**1.04 (0.39, 1.7)**	**0.002**
Drug Use	---	---

**Table 3:** Effect of each health behavior on the reported decrease in physical health during the pandemic between individuals who often/very often compared to rarely/never engaged in health behaviors. Results are predicted means and 95% confidence intervals from linear model analysis adjusted for age, gender, worked in patient care, had school aged children, near high-risk relatives and in high-risk group. Negative values indicate a reduction in psychological well-being decline while positive values indicate an increase in physical health decline.

Health Behavior	Estimate (95% CI)	p-value
Exercise	−0.75 (−1.28, −0.22)	**0.006**
Healthy Eating	−0.81 (−1.44, −0.19)	**0.011**
Getting Enough Sleep	−0.81 (−1.38, −0.25)	**0.005**
Mindfulness or Relaxation Exercises	−0.12 (−0.77, 0.53)	0.709
Focusing on Gratitude and Positive Things	−0.75 (−1.39, −0.12)	**0.02**
Engaging in Pleasurable Activities	−1.20 (−1.75, −0.65)	**<.0001**
Getting Social Support	−1.06 (−1.67, −0.46)	**0.001**
Tobacco Use	1.24 (−0.18, 2.66)	0.086
Alcohol Use	0.18 (−0.44, 0.81)	0.561
Drug Use	---	---

On the other hand, we found that six health behaviors were associated with the degree to which participant’s physical health declined. In comparing self-reported change in physical health during the pandemic, individuals who often/very often engaged in these specific health behaviors: *exercise, healthy eating, adequate sleep, engaging in pleasurable activities, focusing on gratitude and positivity, and getting social support*, experienced a significantly smaller decline in physical health compared to those who rarely/never engage in these behaviors. (Table 3). Negative health behaviors (tobacco and alcohol use) did not have a significant effect on the degree of decline in physical health.

**Fig 6:**
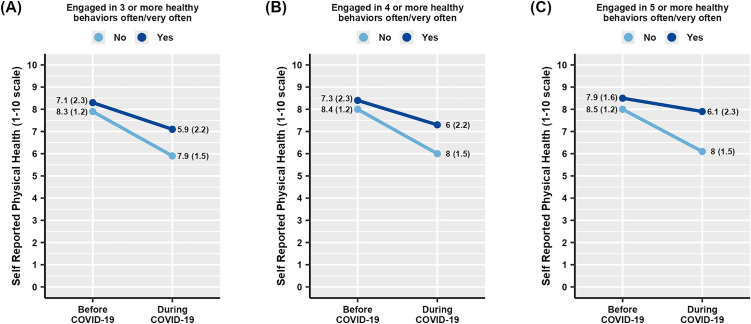
Average self-reported physical health ratings before or during the COVID-19 pandemic among individuals who engaged during the pandemic in more than 3 (A), 4 (B), or 5 (C) positive health behaviors (dark blue lines) compared to those who did not (light blue lines). Values are presented as mean (standard deviation).

Lastly, we examined if engaging in multiple positive health behaviors was related to the decline in psychological well-being or physical health. Individuals who engaged in ≥3, ≥ 4, or ≥5 positive health behaviors had a higher self-rated physical health both prior to and during the pandemic (Fig 6).

When controlling for age, gender and other potential confounders we found that with each additional health behavior the rate of decline in overall physical health decreased by 0.22 points (p = 0.000, 95% CI −0.35, −0.10) (Table 4). Interestingly, males exhibited significantly (p = 0.005) lower rate of decline in physical health with each additional health behavior than females. In contrast, there was no significant association between engaging in multiple health behaviors and the rate of decline in psychological well-being.

**Table 4:** Association between the sum of health behaviors and decrease in physical health from before to during the pandemic. Results are from linear model analysis adjusted for age, gender and additional covariates. Negative values indicate a reduction in physical health decline while positive values indicate an increase in physical health decline.

	Estimate (95% CI)	p-value
Sum of healthy behaviors	−0.22 (−0.35, −0.10)	<0.001
Age		
18–24 years	Reference	
25–34 years	0.19 (−0.85, 1.24)	0.717
35–44 years	0.34 (−0.78, 1.47)	0.551
45–54 years	−0.19 (−1.29, 0.90)	0.733
55–64 years	0.40 (−0.70, 1.49)	0.476
65–74 years	−0.93 (−2.50, 0.65)	0.250
Gender		
Female	Reference	
Male	−0.85 (−1.44, −0.26)	0.005
Other	−1.24 (−5.46, 2.99)	0.566
Patient Care		
No	Reference	
Yes	0.18 (−0.30, 0.67)	0.458
Have school aged children		
No	Reference	
Yes	−0.23 (−0.81, 0.35)	0.432
Related high risk people around		
No	Reference	
Yes	0.53 (−0.01, 1.07)	0.056
In high-risk group		
No	Reference	
Yes	0.31 (−0.26, 0.87)	0.289

## Discussion

Consistent with previous studies, healthcare workers reported a high levels of work related stress, low quality of their personal life, and low levels of psychological well-being and physical health during the COVID-19 pandemic [3,4,11,12]. In this study, we examined if engaging in certain health behaviors might affect the negative impact of the pandemic on the physical health and psychological well-being of healthcare workers. To our knowledge, this is the first study to examine how engaging in a range of positive or negative health behaviors in a real-world setting could potentially mitigate or exacerbate the rate of decline in physical and psychological well-being during COVID-19.

*Engaging in pleasurable activities* was the only health behavior in our study that showed a potential protective effect against the rate of decline in psychological well-being during the pandemic. The beneficial effect of engaging in pleasurable activities on mental health has been demonstrated in other studies [14–18]. It could be argued that these activities serve as a positive distraction from stress and have a restorative effect, enhancing coping and maintaining mental health during periods of high stress and uncertainty [19]. When people cease to engage in pleasurable activities, the quality of their life decreases and they are more likely to experience negative psychological sequelae, including depressed mood. Many psychological interventions for depression include scheduling pleasurable activities to counter the decline in psychological health when people are left with few things to look forward to [20]. Other positive health behaviors (e.g., exercise, sleep) did not show a protective effect on psychological well-being, which may be explained by the fact that pleasurable activities provide more immediate or direct emotional relief from stress, whereas the benefits of exercise and sleep are often more gradual or less noticeable during periods of acute, ongoing stress.

In contrast to psychological well-being, our assessment of factors affecting physical health revealed that *engaging in pleasurable activities, focusing on gratitude and positive things, getting social support, exercise, healthy eating, and getting enough sleep,* all had a possible protective effect on the rate of decline in physical health. It is unclear why these health behaviors appear to counteract the decline in physical health during the pandemic but not psychological well-being. A possible explanation is that there is a two-way relationship between health behaviors and health status. People who were feeling physically well may have been better positioned to engage in an array of healthy behaviors and coping strategies whereas the same may not have been true for those who were psychologically doing well.

We also found that engaging in these positive health behaviors during the pandemic was associated with better self-rated of psychological well-being and physical health during the pandemic with one exception. Surprisingly, regular practice of *mindfulness or relaxation exercises* was not associated with significantly higher levels of psychological well-being or physical health. This contrasts with other studies that describe powerful benefits of mindfulness and relaxation exercises, including among populations exposed to high levels of stress [21–26]. This finding could be due to the fact that relatively few people in our sample (14.8%) reported engaging often/very often in *mindfulness or relaxation exercises*. It is also possible that those who reported practicing mindfulness and relaxation exercises may not have understood what those exercises entailed. We did not assess whether our participants had received any formal training in mindfulness or relaxation exercises. Studies demonstrating the effectiveness of mindfulness exercises typically involve rigorous and formalized mindfulness interventions consisting of 4–10 weekly sessions lasting 1 ½ to 2 ½ hours each, coupled with daily practice between sessions [21]. Another study during the COVID-19 pandemic found that the effectiveness of mindfulness practice was dependent on the amount of previous structured training coupled with the frequency of current practice [27].

Alcohol use was the most common negative health behavior reported in our study, consistent with the reported increase in alcohol consumption in the United States during the COVID-19 pandemic [28]. In our study, healthcare workers who reported using alcohol to cope during the pandemic had lower self-rating of physical health and psychological wellbeing during the pandemic. A similar finding was observed among those who reported tobacco use. This is consistent with studies that reported increase in tobacco use during the COVID pandemic and its association with lower life satisfaction in health professionals [29,30]. Healthcare workers in our study did not report drug use as a means of coping, (except 1.9% reported drug use sometimes), which is not consistent with the increase in controlled substance or illicit drug use during the pandemic in the larger population [31]. We suspect that healthcare workers are less likely to misuse prescription or illicit drugs in general given the nature of their profession. It is also possible that there was underreporting of drug use in this study due to the fear of employment repercussions. Even though the survey was collected anonymously, participants were recruited through their place of employment. While higher engagement in negative health behaviors can cause a harmful effect on one’s health during a stressful period, the other possibility here is that healthcare workers increase their engagement in negative health behaviors as an attempt to cope with stress.

Participants who frequently engaging in multiple positive healthy behaviors rated both their psychological well-being and physical health higher during the pandemic, and this effect increased with the number of healthy behaviors. Others have also described the additive effect of multiple health behaviors on physical and psychological well-being [32]. This is also consistent with findings from a sample of Portuguese nurses in which better mental health was associated with eating healthy, physical activity, rest between shifts, maintaining social contacts, verbalizing feelings, and spending less time searching for COVID-19 information [33]. A similar study out of Poland concluded that nurses who engaged in positive health behaviors had better quality of life across domains [32]. Given the potential impact of individual health behaviors on the rate of decline on physical health and psychological well-being, it was not surprising to find that there was an additive effect of multiple health behaviors on the rate of decline on physical health but not on psychological well-being.

### Limitations

Our study has several limitations. The accuracy of self-report measures may be affected by biases such as self-deception, inaccurate memories, and motivated forgetfulness [34,35]. The survey assessed health behaviors and coping strategies during the pandemic but did not assess baseline rates of these behaviors. Therefore, it is unclear if the reported health behaviors and coping strategies were adopted during the pandemic or were pre-existing and maintained, or how changes in these behaviors impacted health and psychological well-being ratings. Regardless, those who were able to either adopt or maintain previously employed positive health behaviors and coping strategies fared better during the pandemic and showed differences in the rate of decline from baseline. During times of stress, others have reported that positive health behaviors and coping strategies tend to decline with an increase in negative behaviors and strategies [36,37]. Hence, we suspect that for most of our participants, the positive behaviors and strategies that they reported were maintained from baseline, likely explaining why they also reported faring better prior to the pandemic as well as during. Further research in this area is warranted to better understand the impact of adding new strategies or maintaining previously employed strategies on physical health and psychological well-being. Additionally, retrospective ratings of pre-pandemic physical health and psychological well-being may been influenced by participants experiences during the pandemic. Finally, The generalizability of these findings is limited by the sample characteristics, as participants were predominantly female, younger in age, and recruited from a single health system. While such demographic patterns are common in healthcare worker studies, they may restrict the applicability of results to broader or more diverse populations.

## Conclusion

This study found that engaging in positive health behaviors and coping strategies during COVID-19 had a beneficial impact on physical and psychological well-being. Our findings suggest that initiating new or maintaining previously established positive health behaviors could possibly have a protective effect on the rate of decline in physical health and, to a lesser extent, on psychological well-being during periods of stress.

In particular, our study demonstrates that *engaging in pleasurable activities* is associated with reduced declines in both physical health and psychological well-being. This finding could inform future workplace wellness policies. Organizations should consider implementing structured programs that encourage participation in enjoyable activities during the workday, such as group exercise sessions, creative workshops, or mindfulness breaks as part of wellness initiatives, especially during times of increased stress.

## Supporting information

S1 Fig The number of COVID-19 patients admitted to the health system during the pandemic with the timing of data collection for the 2 study phases. (TIF)

S2 Fig The study survey. (PDF)

S3 Fig Changes in personal life reported by study participants during the pandemic compared to before. (TIFF)

S4 Fig Changes in work life reported by study participants during the pandemic compared to before. (TIFF)

S5 Fig Changes in emotions reported by participants during the pandemic compared to before. (TIFF)
