# Supplementary figures and images for: Association of health behaviors with healthcare workers’ physical and psychological well-being: Learning from the COVID-19 pandemic
Source: PLoS One. 2025 Oct 31;20(10):e0334752. doi: 10.1371/journal.pone.0334752 (PMC12578231; doi:10.1371/journal.pone.0334752)

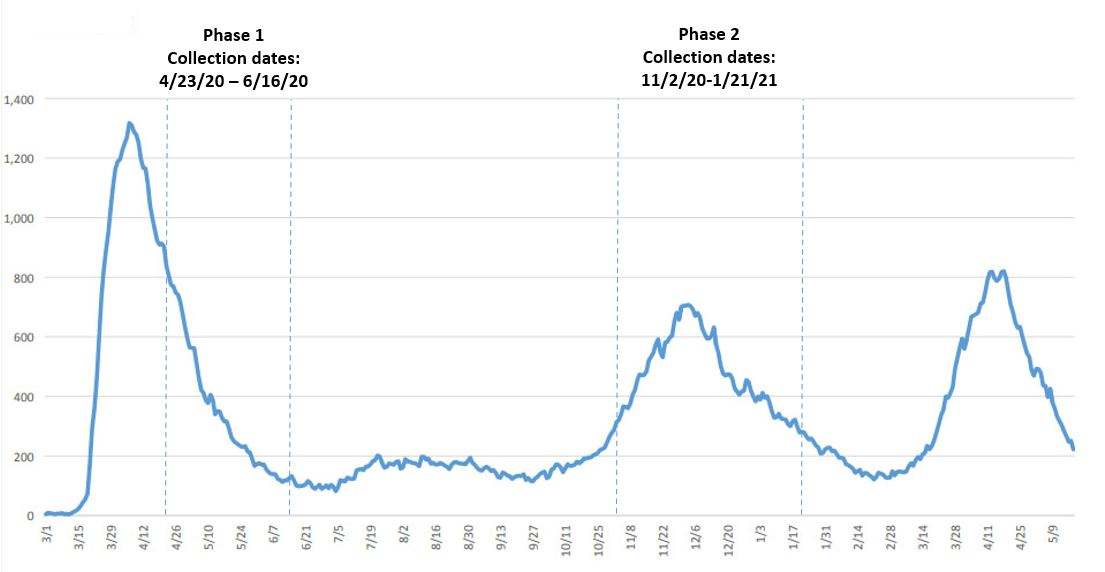

Supplement: S1 Fig — (TIF) [file pone.0334752.s001.tif]

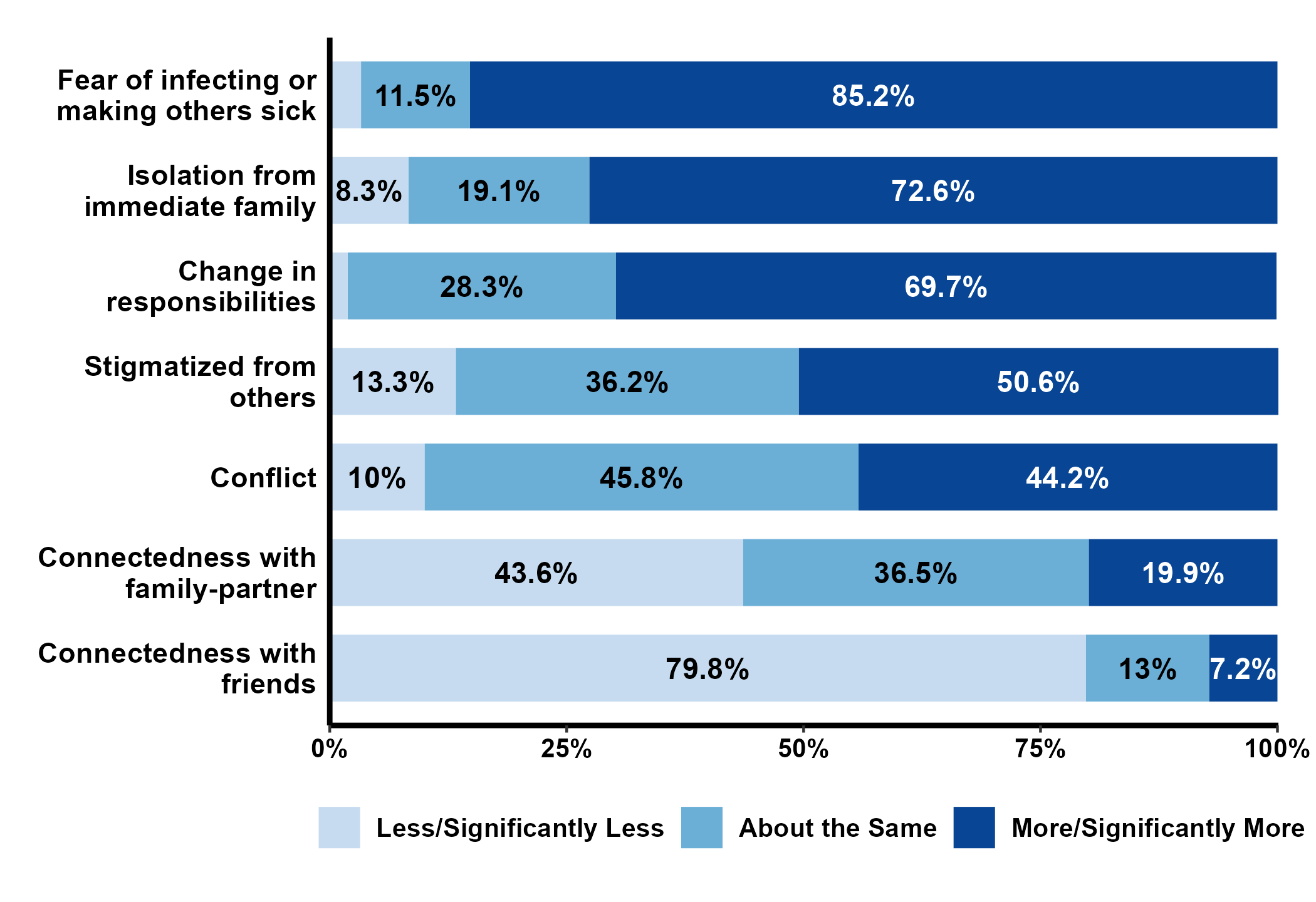

Supplement: S3 Fig — (TIFF) [file pone.0334752.s003.tiff]

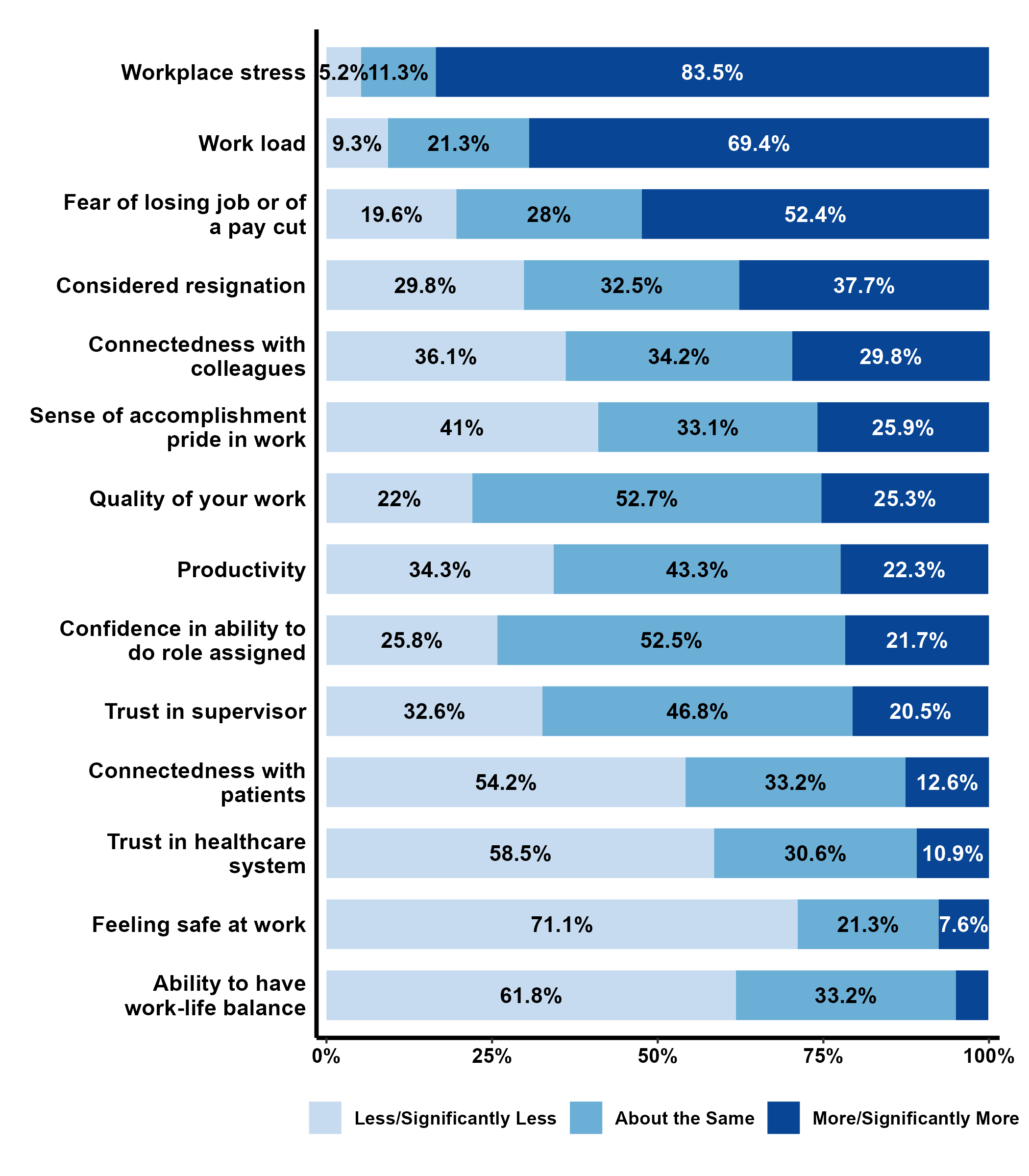

Supplement: S4 Fig — (TIFF) [file pone.0334752.s004.tiff]

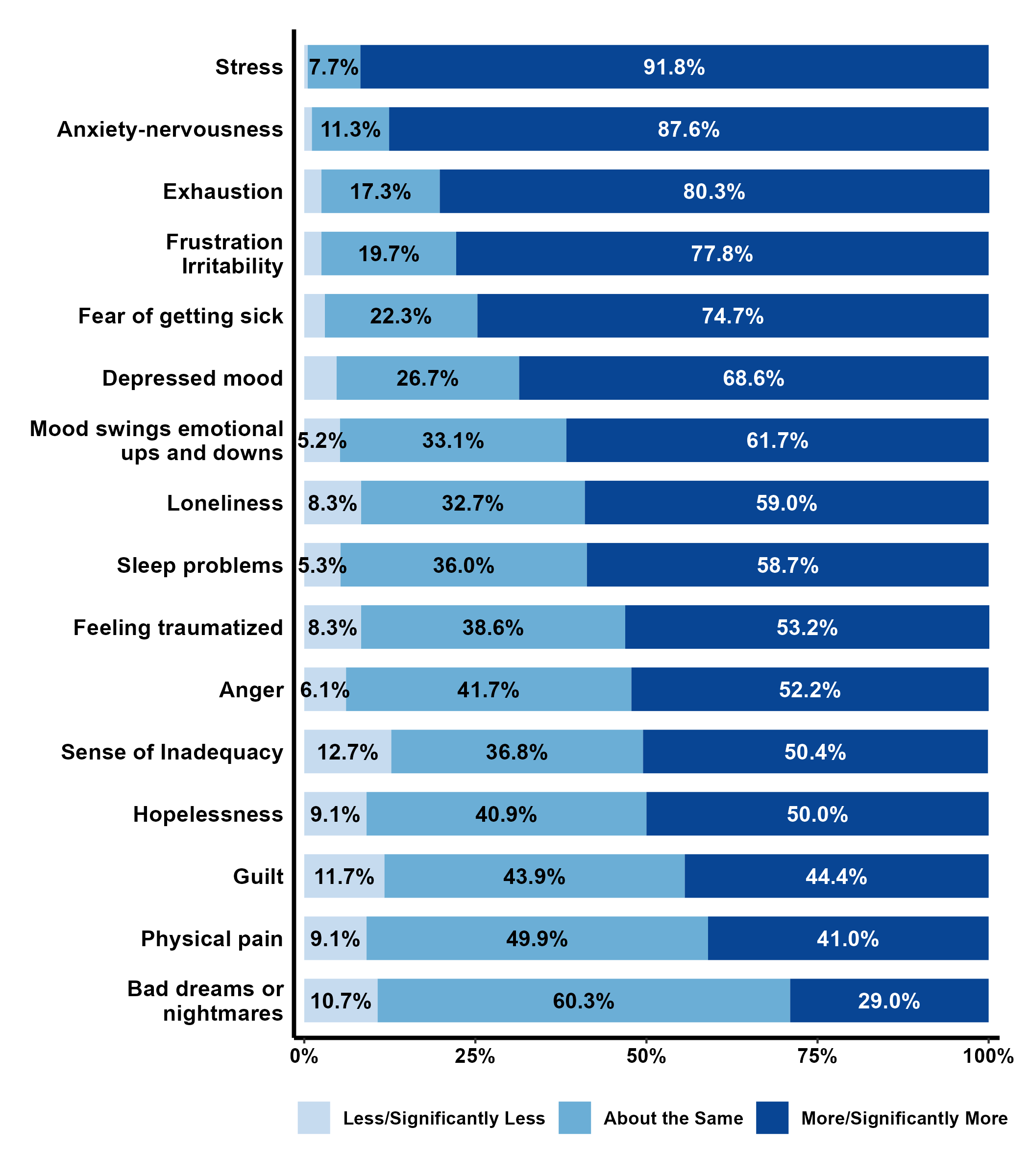

Supplement: S5 Fig — (TIFF) [file pone.0334752.s005.tiff]
