# Supplementary material for: Association of health behaviors with healthcare workers’ physical and psychological well-being: Learning from the COVID-19 pandemic
Source: PLoS One. 2025 Oct 31;20(10):e0334752. doi: 10.1371/journal.pone.0334752 (PMC12578231; doi:10.1371/journal.pone.0334752)
Supplement: S2 Fig — (PDF) [file pone.0334752.s002.pdf]

| **Please rate each item comparing your WORK LIFE Now to before COVID-19 pandemic** | **Significantly less** | **Less** | **About the same** | **More** | **Significantly more** |
|---|---|---|---|---|---|
| Workload (amount of work you had to do) | | | | | |
| Quality of your work | | | | | |
| Connectedness with colleagues | | | | | |
| Connectedness with patients | | | | | |
| Productivity (amount of work you were able to do) | | | | | |
| Sense of accomplishment/pride in work | | | | | |
| Confidence in ability to do role assigned | | | | | |
| Feeling safe at work | | | | | |
| Workplace stress | | | | | |
| Fear of losing job or of a pay cut | | | | | |
| Trust in supervisor | | | | | |
| Trust in healthcare system | | | | | |
| Considered resignation | | | | | |
| Ability to have work/life balance | | | | | |
| | | | | | |
| Comments about impact of COVID-19 on your work life: | | | | | |
| **Please rate each item comparing your PERSONAL LIFE Now to before COVID-19 pandemic** | **Significantly less** | **Less** | **About the same** | **More** | **Significantly more** |
| Connectedness with family/partner | | | | | |
| Connectedness with friends | | | | | |
| Conflict | | | | | |
| Isolation from immediate family | | | | | |
| | | | | | |

| | | | | | |
|---|---|---|---|---|---|
| Stigmatized (viewed negatively) from others | | | | | |
| Fear of infecting or making others sick | | | | | |
| Responsibilities | | | | | |
| Comments about impact of COVID-19 on your personal life: | | | | | |
| **Please rate each item comparing your EMOTIONS AND HEALTH Now to before COVID-19 pandemic** | **Significantly less** | **Less** | **About the same** | **More** | **Significantly more** |
| Stress | | | | | |
| Anxiety/nervousness | | | | | |
| Depressed mood | | | | | |
| Anger | | | | | |
| Frustration/Irritability | | | | | |
| Mood swings, emotional ups and downs | | | | | |
| Exhaustion | | | | | |
| Sense of Inadequacy | | | | | |
| Feeling traumatized | | | | | |
| Guilt | | | | | |
| Hopelessness | | | | | |
| Loneliness | | | | | |
| Sleep problems | | | | | |
| Bad dreams or nightmares | | | | | |
| Fear of getting sick | | | | | |
| Physical pain | | | | | |
| **OVERALL HEALTH AND WELL-BEING: Please rate each item on a scale from 1 to 10 (1 = very poor, 10 = very good)** | | | | | |
| Your overall psychological well-being **BEFORE** COVID-19: (drop down box with numbers 1 to 10) | | | | | |
| Your overall psychological well-being **DURING** COVID-19: (drop down box with numbers 1 to 10) | | | | | |
| Your overall psychological well-being **NOW**: (drop down box with numbers 1 to 10) | | | | | |
| Your overall physical health **BEFORE** COVID-19: (drop down box with numbers 1 to 10) | | | | | |

| | | | | | |
|---|---|---|---|---|---|
| Your overall physical health **DURING** COVID-19: (drop down box with numbers 1 to 10)<br>Your overall physical health **NOW:** (drop down box with numbers 1 to 10) | | | | | |
| | | | | | |
| Comments about impact of COVID-19 on your emotions and health: | | | | | |
| **Health behaviors and coping strategies: Please rate how often you have engaged in the following health behaviors or coping strategies in the last 6 months.** | **Never** | **Rarely** | **Sometimes** | **Often** | **Very Often** |
| Exercise | | | | | |
| Healthy eating | | | | | |
| Getting enough sleep | | | | | |
| Mindfulness/relaxation exercises | | | | | |
| Focusing on gratitude and positive things | | | | | |
| Engaging in pleasurable activities | | | | | |
| Getting social support | | | | | |
| Nicotine/tobacco use | | | | | |
| Alcohol use | | | | | |
| Drug use | | | | | |
| | | | | | |

In the last 6 months did you…

- Seek services from a mental health professional to help you cope? Yes No
- Start or change medication for anxiety or depression to help you cope? Yes No
- Use employee wellness services designed to ease the stress (e.g, EAP, BWell, chaplain, group support sessions)? Yes No

Comments about your health behaviors and coping strategies:
