# Supplementary material for: Association of health behaviors with healthcare workers’ physical and psychological well-being: Learning from the COVID-19 pandemic
Source: PLoS One. 2025 Oct 31;20(10):e0334752. doi: 10.1371/journal.pone.0334752 (PMC12578231; doi:10.1371/journal.pone.0334752)
Supplement: Attachment — Submitted filename: PONE-D-24-07740-Review.pdf [file pone.0334752.s007.pdf]

Review of the manuscript: "**Association of Health Behaviors with Healthcare Workers' Physical and Psychological Well-being: Learning from the COVID-19 Pandemic**"

I am grateful for the chance to contribute to the peer-review process of this manuscript.

The general aim of the work is clear: to see if there is an association between health behaviors during the pandemic (or rather, the second wave of the pandemic) and self-reported physical and psychological health. However, there is a certain ambiguity in the objectives, given that analyzes referring to during the pandemic alternate with analyzes in which changes from before to during the pandemic are evaluated. I suggest clarifying clearly in the Introduction what scientific questions the authors aim to answer.

Furthermore, many details of the study and data analysis are omitted and it is therefore difficult to understand the significance of the results.

Major remarks

Introduction

1. Psychological health (PH) has not been defined. It seems to me that in the manuscript this term is used as a synonym for psychological well-being (see the title) or stress (for example in the second and third paragraphs of the Introduction or in the Discussion, where numerous references to stress are found) or, to a lesser extent, to mental health. The authors must give their definition of the construct, and the related bibliographical references.

2. Burnout is the big elephant in the room. Given the relevance that burnout has in the literature on the psychological well-being of healthcare workers, the authors should try to explain why they did not refer to this construct and what advantages their approach brings instead.

3. The authors claim that "The effectiveness of health behaviors on coping with stress is not well assessed in the literature". Soon after, the authors also claim that "it is unclear how health behaviors counteract the negative effects of stress". These sentences seem too drastic to me. For example, studies evaluating either the association or the effectiveness of physical exercise as a coping strategy are truly numerous. Authors should explore the literature further in this Introduction, to better contextualize their study.

Setting and study population

4. I suggest to revise this subsection by firstly describing the overall study and then specifying the 2 different phases. I also suggest indicating if and how the first phase of the study was used to develop the questionnaire. The bibliographical reference is useful, but some explanation given explicitly would facilitate the reader in understanding the study. Moreover, in the third line of the Data collection section it is stated that at the website participants could "to answer survey questions, submit an audio recording or do both". Are the audios shown here the ones referred to as "phase 1"? In this case it would seem that the two phases went in parallel. This point needs to be clarified.

Furthermore, I would suggest to provide a few more details about the "large healthcare system" under study in terms of both size and impact of the pandemic on it.

Data collection

In this sub-section, authors mention that "A list of institutional support resources was provided in case participants were in distress". How does this relate to the study?

Survey

5. The description of the questionnaire must be expanded, specifying the measures considered, the questions asked, the answers to the closed ones, etc. At the current state of the section, it is also difficult to interpret the results part and understand the analyzes performed. It must also be specified for which of the variables the retrospective (pre-pandemic) evaluation was also requested.

6. If I understood correctly, the retrospective evaluation was requested for physical health and psychological health but not for health behaviors. It's correct? So we don't know if the pandemic has changed the frequency of engaging in healthy habits, right? I suggest clarifying this point.

Since authors often refer to the concept of coping strategy, then I would expect to clearly know from respondents whether a health behavior has been adopted as a coping strategy (i.e., an effort to reduce or adapt to stressful conditions and emotional distress associated in the COVID situation). pandemic) or if it was already an established habit before the pandemic.

If my hypothesis is correct, I suggest clearly explaining the rationale for the authors' choice (which, beyond review, does not seem entirely convincing to me: what additional information does this study bring compared to a traditional cross-sectional study to associate health behaviors and physical/psychological health during the pandemic?).

I would also suggest indicating within the limitations of the study that this choice prevents the assessment of the association between changes in the frequency of health behaviors and changes in physical health and psychological health. Changes in the frequency of health behaviors rather than engagement or not engagement during the pandemic, could be associated to the changes in physical/psychological health rates. Furthermore, knowing whether the health behavior (either positive or negative) was adopted during the pandemic would tell us something about whether it was chosen as a coping strategy versus whether it was an already established habit.

Statistical analysis

7. I suggest to revise this section. First, the authors need to specify a list of statistical questions they aimed at answering. Second, they should better clarify the methods used for each analysis. For example, there is no indication of the tests used in Figures 4 and 5. The authors refer to a "planned comparison," but this phrase does not explain how the comparison was made.
In particular, the use of linear models must be clarified very well. The change in the physical/psychological health is a categorical rather than a continuous variable and this compromises the reliability of results. In my experience, with this kind of data the residuals rarely show Gaussian trends and pass the Shapiro-Wilks test.

Results

8. Regarding the analyzes carried out on multiple positive health behaviors. In my opinion, having conducted three analyzes separately does not allow us to understand whether there is a ceiling effect on the accumulation of healthy behaviors. I suggest considering a single model with a single covariate indicating the number of health behaviors adopted.

Discussion.

9. The Discussion is affected by the same critical issues identified for the Introduction and should be revised accordingly, taking into account possible changes in the results.

Minor remarks

Setting and study population

1. The reference to Figure 1 in the fourth line does not seem relevant to the sentence that precedes it. A bibliographical reference would be useful.

Results

2. In Tables 2 and 3, “estimate” means the regression coefficient? Reading in the text, it would almost seem like the fitted (predicted) value.

There are several references related to the mental health of HCWs which could be updated with more recent articles.
