# Supplementary material for: Association of health behaviors with healthcare workers’ physical and psychological well-being: Learning from the COVID-19 pandemic
Source: PLoS One. 2025 Oct 31;20(10):e0334752. doi: 10.1371/journal.pone.0334752 (PMC12578231; doi:10.1371/journal.pone.0334752)
Supplement: Attachment — Submitted filename: PLOS ONE response to reviewer 3 11 25.docx [file pone.0334752.s008.docx]

Anna Rachel Conolly, PhD, MSc, PG Dip, BA (hons)
Academic Editor
PLOS ONE

Dear Dr. Conolly,

Thank you for the opportunity to revise our manuscript, “Association of Health Behaviors with Healthcare Workers’ Physical and Psychological Well-being: Learning from the COVID-19 Pandemic” based on feedback from your reviewer. Their feedback regarding ways to improve the quality of our manuscript is much appreciated. We believe we have adequately addressed each of their comments as described below:

**Major remarks**

***Introduction***

1. **Psychological health (PH) has not been defined. It seems to me that in the manuscript this term is used as a synonym for psychological well-being (see the title) or stress (for example in the second and third paragraphs of the Introduction or in the Discussion, where numerous references to stress are found) or, to a lesser extent, to mental health. The authors must give their definition of the construct, and the related bibliographical references.**

- We replaced the term “psychological health” with “psychological well-being” throughout the manuscript for consistency and better clarity and to align with the wording that was used in the survey.
- We intentionally used this broad term to capture concepts beyond the absence of a mental health disorder. As with physical health, in our study survey we left the term up for interpretation to each participant, (i.e., asking them to rate each of the following: their overall physical health and their overall psychological well-being both before and during COVID). This item in the survey followed a section asking participants to rate a list of emotions comparing how they experienced them during COVID to prior to COVID. A copy of survey has been included as a supplemental figure for further clarification.

2. **Burnout is the big elephant in the room. Given the relevance that burnout has in the literature on the psychological well-being of healthcare workers, the authors should try to explain why they did not refer to this construct and what advantages their approach brings instead.**
   - We agree that burnout is an important construct and related to our study topic. Most commonly, burnout refers to a response to a negative work environment. In the first part of this study, we described new meaningful experiences reported by healthcare workers during the pandemic and we did not want the survey to only focus on the negative work environment only. *(Lackman Zeman, L., Roy, S., Surnis, P. P., Wasserman, J. A., Duchak, K., Homayouni, R., & Mulhem, E. (2023). Paradoxical experiences of healthcare workers during COVID-19: a qualitative analysis of anonymous, web-based, audio narratives. International Journal of Qualitative Studies on Health and Well-Being, 18(1). https://doi.org/10.1080/17482631.2023.2184034)*
   - The COVID-19 pandemic was unique in that in addition to directly impacting the work environment, it also directly impacted healthcare workers’ personal lives. We wanted to learn how both components of work and personal life changed in response to the pandemic, how these changes related to changes in emotions and health, what types of health behaviors and coping strategies healthcare workers employed, and how these behaviors and coping strategies were related to rate of decline in physical health status and psychological well-being. By adding a measure of burnout, we were concerned about making our survey too long.
3. **The authors claim that “The effectiveness of health behaviors on coping with stress is not well assessed in the literature”. Soon after, the authors also claim that “it is unclear how health behaviors counteract the negative effects of stress”. These sentences seem too drastic to me. For example, studies evaluating either the association or the effectiveness of physical exercise as a coping strategy are truly numerous. Authors should explore the literature further in this Introduction, to better contextualize their study.**
   - We removed these statements from the manuscript.

***Setting and study population***

4. **I suggest to revise this subsection by firstly describing the overall study and then specifying the 2 different phases. I also suggest indicating if and how the first phase of the study was used to develop the questionnaire. The bibliographical reference is useful, but some explanation given explicitly would facilitate the reader in understanding the study. Moreover, in the third line of the Data collection section it is stated that at the website participants could “to answer survey questions, submit an audio recording or do both”. Are the audios shown here the ones referred to as "phase 1"? In this case it would seem that the two phases went in parallel. This point needs to be clarified.**
   - We have rewritten this section to provide better clarification and added a figure S1, that shows the number of COVID-19 patients hospitalized in our health system during the study period.

**Furthermore, I would suggest to provide a few more details about the “large healthcare system” under study in terms of both size and impact of the pandemic on it.**

We added the following: “This study took place in a large healthcare system in Southeast Michigan, consisting of eight hospitals with 3,375 beds and 155 outpatient sites.”

***Data collection***

**In this sub-section, authors mention that “A list of institutional support resources was provided in case participants were in distress”. How does this relate to the study?**

- This was related to an earlier part of this study and is not related to the survey part of the study we are reporting on here. We omitted this sentence.

***Survey***

5. **The description of the questionnaire must be expanded, specifying the measures considered, the questions asked, the answers to the closed ones, etc. At the current state of the section, it is also difficult to interpret the results part and understand the analyzes performed. It must also be specified for which of the variables the retrospective (pre-pandemic) evaluation was also requested.**
   - This section of the manuscript has been expanded to include more details about the survey. A copy of the survey was included as a supplementary figure.
6. **If I understood correctly, the retrospective evaluation was requested for physical health and psychological health but not for health behaviors. It's correct? So we don't know if the pandemic has changed the frequency of engaging in healthy habits, right? I suggest clarifying this point.**
   - This is correct and is noted as a limitation in the limitation section of the manuscript and elaborated on in the revision. “The survey assessed health behaviors and coping strategies during the pandemic but did not assess baseline rates of engaging in health these behaviors……..”

**Since authors often refer to the concept of coping strategy, then I would expect to clearly know from respondents whether a health behavior has been adopted as a coping strategy (i.e., an effort to reduce or adapt to stressful conditions and emotional distress associated in the COVID situation).pandemic) or if it was already an established habit before the pandemic.**

- We agree that this is a limitation of the study and is discussed in the limitation section of the manuscript. We asked our participants the following “please rate how often you have engaged in the following health behaviors or coping strategies during COVID-19” which we clarified in the methods section. In hindsight, it would have been useful to add a column in the survey so the participants can check if these health behaviors were the same, increased or decreased compared to before the pandemic.
- We elaborated on this as a limitation of this study and modified the language in our conclusion. Regardless of whether or not the health behaviors and coping strategies endorsed were new or previously established and maintained, we believe the findings and implications are still significant. Our primary aim was not to learn what new strategies people employed to cope but rather to understand the association between what they did do and how that was associated with ratings of psychological well-being and health status during COVID and how their strategies were associated with the rate of decline from baseline for each individual.

**If my hypothesis is correct, I suggest clearly explaining the rationale for the authors’ choice (which, beyond review, does not seem entirely convincing to me: what additional information does this study bring compared to a traditional cross-sectional study to associate health behaviors and physical/psychological health during the pandemic?).**

- As noted above, we agree that an understanding of baseline rates of coping and health behaviors would be useful, and this was discussed in details in the limitation section. However, we still believe that our findings relevant to how maintained or newly employed coping strategies and health behaviors may have mitigated the rate of decline in physical health and psychological well-being during the pandemic. These finding are worthy of noting and of further exploration.

**I would also suggest indicating within the limitations of the study that this choice prevents the assessment of the association between changes in the frequency of health behaviors and changes in physical health and psychological health. Changes in the frequency of health behaviors rather than engagement or not engagement during the pandemic, could be associated to the changes in physical/psychological health rates. Furthermore, knowing whether the health behavior (either positive or negative) was adopted during the pandemic would tell us something about whether it was chosen as a coping strategy versus whether it was an already established habit.**

- We agree with the reviewer and have elaborated on this in the limitations section.

***Statistical analysis***

7. **I suggest to revise this section. First, the authors need to specify a list of statistical questions they aimed at answering. Second, they should better clarify the methods used for each analysis. For example, there is no indication of the tests used in Figures 4 and 5. The authors refer to a “planned comparison,” but this phrase does not explain how the comparison was made. In particular, the use of linear models must be clarified very well. The change in the physical/psychological health is a categorical rather than a continuous variable and this compromises the reliability of results. In my experience, with this kind of data the residuals rarely show Gaussian trends and pass the Shapiro-Wilks test.**

- We apologize for any confusion and have significantly rewritten this section. In addition, we did non-parametric tests instead of parametric tests to address the concerns about non-normality. Results and discussion changed to reflect the new results.

***Results***

8. **Regarding the analyzes carried out on multiple positive health behaviors. In my opinion, having conducted three analyzes separately does not allow us to understand whether there is a ceiling effect on the accumulation of healthy behaviors. I suggest considering a single model with a single covariate indicating the number of health behaviors adopted.**

- We believe the reviewer is referring to figure 6 when they refer to “3 analyses.”  The reviewer seems to imply that it isn’t adding much value.  We agree that presenting the 3 analyses may not show a ceiling effect, but we believe the figure provide a visual representation of the change in psychological and physical wellbeing related to the number of health behaviors reported by participants in the study.

***Discussion***

9. **The Discussion is affected by the same critical issues identified for the Introduction and should be revised accordingly, taking into account possible changes in the results.**
   - We have revised accordingly.

**Minor remarks**

***Setting and study population***

1. **The reference to Figure 1 in the fourth line does not seem relevant to the sentence that precedes it. A bibliographical reference would be useful.**
   - Reference corrected

***Results***

**2. In Tables 2 and 3, “estimate” means the regression coefficient? Reading in the text, it would almost seem like the fitted (predicted) value.**

- The figures description updated, added: “Results are predicted means and 95% confidence intervals from linear model analysis adjusted for age, gender, worked in patient care, had school aged children, near high-risk relatives and in high-risk group”

**There are several references related to the mental health of HCWs which could be updated with more recent articles.**

- References reviewed and we believe they are up to date. We added additional recent references
