# Supplementary material for: Association of health behaviors with healthcare workers’ physical and psychological well-being: Learning from the COVID-19 pandemic
Source: PLoS One. 2025 Oct 31;20(10):e0334752. doi: 10.1371/journal.pone.0334752 (PMC12578231; doi:10.1371/journal.pone.0334752)
Supplement: Attachment — Submitted filename: Reviewers comment 2.docx [file pone.0334752.s009.docx]

Reviewer #1: First of all, I would like to thank the authors for their extensive review and detailed responses to my comments. I have just a few small comments to make, to conclude.

**Thank you**

1. Section "Statistical analysis": Please, move the sentence "Residual versus fits plots and quantile

quantile plots for all linear models were inspected to verify the model assumptions were reasonable. All analyses were performed using R Statistical Software (v4.2.2; R Core Team 2022)." to the subsession "Association Between Change in Physical Health and Psychological Well-Being and Health Behaviors", where linear models are introduced

**Sentence moved as recommended**

2. Table 1. Please, note that the Fisher test for tables larger than 2x2 is called the Fisher-Freeman-Halton test (Freeman G.H. and Halton J.H. (1951), Note on an exact treatment of contingency, goodness of fit and other problems of significance. Biometrika 38:141-149).

**Changed Fisher exact to Fisher-Freeman-Halton**

3. The opening sentence of the discussion: This study found that healthcare workers who regularly engaged in positive health behaviors and coping strategies during COVID-19 did better psychologically and physically **both** before and during COVID-19" is not supported by the data. In fact, engagin in health behaviors and coping strategies during COVID-19 was associated to CHANGES in physical health adn psychologicalwell-being. Please, refrase this sentence.

**Sentence changed to “****engaging in positive health behaviors and coping strategies during COVID-19 had a positive impact on physical and psychological well-being”**

4. Figure 6: I suggest completing the caption "Engaged ...." by adding **during the pandemic**. I also suggest moving it and the "No/Yes" legend **above** each of the three graphs. In its current position, the caption seems to suggest that engagement was detected both before and during the pandemic. Below each graph, simply indicate "self-reported physical health" or something like this.

**Added “during the pandemic” to the caption.**

**We have yes/no at the bottom of each graph.**

**Changed “physical health” to “self-reported physical health” in the caption.**

Reviewer #2: 1. The manuscript reads much better now—thank you for addressing the previous round of comments. The flow of the introduction and the rationale is clearer.

**Thank you**

2. I appreciate the effort in the new data analyses. Including adjusted linear models for each health behavior is a solid addition and strengthens the statistical rigor.

**Thank you**

3. The distinction between physical and psychological well-being outcomes is clearly articulated, although some transitions between these concepts in the discussion could still be tightened.

**Please provide specific areas in the discussion where this can be done**

4. I still feel the lack of baseline data on health behaviors (i.e., pre-pandemic) is a notable limitation—perhaps this could be more explicitly acknowledged upfront in the limitations section, not just at the end.

**This issue is discussed clearly in the limitation section, and it occupies most of the section, we don’t feel that moving it up couple lines will make any significant difference.**

5. The finding that only pleasurable activities had a protective effect on psychological well-being is intriguing. It might be worth briefly discussing why other commonly cited interventions (e.g., sleep, exercise) didn’t have similar effects here.

**We already covered this point in the discussion “It is unclear why these health behaviors appear to counteract the decline in physical health during the pandemic but not psychological well-being. A possible explanation is that there is a two-way relationship between health behaviors and health status. People who were feeling physically well may have been better positioned to engage in an array of healthy behaviors and coping strategies whereas the same may not have been true for those who were psychologically doing well.”**

6. The explanation about why mindfulness/relaxation exercises may not have shown significant results is well-reasoned. Good job offering a plausible rationale based on lack of formal training.

**Thank you**

7. There’s a lot of useful data in the figures, but some are dense. Simplifying captions and highlighting the key takeaway from each figure would help guide the reader better.

**We made changes to captions as recommended by reviewer 1. Please be more specific about changes you recommend.**

8. The manuscript could benefit from a light copyedit for grammar and flow. Some sections still contain redundant phrases or awkward syntax (e.g., “did reported better”).

**We reviewed the manuscript and did not find “did reported better” phrase.**

9. On generalizability—can you comment on how representative this sample is of U.S. healthcare workers? The demographics lean heavily female and young, which is common but may still limit wider applicability.

**The sample collected from one large health system in Southeast Michigan, which is a factor in limiting generalizability.**

10. The conclusion is appropriately cautious, though I’d suggest briefly noting how these findings could inform specific workplace wellness policies moving forward, beyond general suggestions.

**Changed the last sentence to: “This finding could inform specific workplace wellness policies moving forward. Interventions should focus on creating mechanisms to increase opportunities for engaging in pleasurable activities, both in the workplace and at home, especially during times of increased stress.”**
