# Supplementary material for: Association of health behaviors with healthcare workers’ physical and psychological well-being: Learning from the COVID-19 pandemic
Source: PLoS One. 2025 Oct 31;20(10):e0334752. doi: 10.1371/journal.pone.0334752 (PMC12578231; doi:10.1371/journal.pone.0334752)
Supplement: Attachment — Submitted filename: PLOS ONE editors comments- reply.docx [file pone.0334752.s010.docx]

Dear Dr. Plos One team,

Thank you again for your careful revisions and responses. I apologize for the earlier oversight — I am currently reviewing many submissions, and I appreciate your patience. After reviewing the manuscript and the latest set of comments, I believe the paper is very close to acceptance and only requires minor revisions to further improve clarity and flow.

**Thank you for your comments, we addressed your recommendations in bold below:**

Please see the remaining points below:

- Move the sentence about residual versus fits plots, quantile–quantile plots, and the use of R software into the subsection where linear models are introduced.
  - **Sentence moved as requested**
- For Table 1, update the terminology to refer to the Fisher–Freeman–Halton test when contingency tables are larger than 2×2.
  - **Added**
- In the Discussion, the opening sentence suggests outcomes were tracked both before and during COVID. Since the data focus on change during the pandemic, please rephrase to avoid overstating.
  - **The opening sentence changed to: Consistent with previous studies from our group and others, healthcare workers reported high levels of work- related stress, low quality of their personal life, and low levels of psychological well-being and physical health during the COVID-19 pandemic (3, 4, 11, 12, 14).**
- For Figure 6, add the phrase “during the pandemic” in the caption, shift the Yes/No legend above each graph, and clarify the x-axis label as “self-reported physical health.”
  - **Changes made to the figure and capture as suggested.**
- The transitions between psychological well-being and physical health in the Discussion could be tightened to improve readability.
  - **We changed the transition sentence to: In contrast to psychological well-being, our assessment of factors affecting physical health revealed that……**
- The limitation regarding the lack of baseline (pre-pandemic) health behavior data should be emphasized more prominently.
  - **We made minor edits, and we feel that this limitation is thoroughly addressed in the limitation section, that discussion constitutes more than two-thirds of that section.**
- Since only pleasurable activities showed a protective effect on psychological well-being, a brief reflection on why other health behaviors (e.g., exercise, sleep) did not have similar effects would strengthen the Discussion.
  - **Added to the discussion “****Other positive health behaviors (e.g., exercise, sleep) did not show a protective effect on psychological well-being, which may be explained by the fact that pleasurable activities provide more immediate or direct emotional relief from stress, whereas the benefits of exercise and sleep are often more gradual or less noticeable during periods of acute, ongoing stress.”**
- The rationale regarding mindfulness/relaxation is clear and well-reasoned; just ensure it remains concise.
  - **Thank you**
- Some figure captions could be simplified and should highlight the main takeaway for each figure.
  - **Changes made to simplify figures captions**
- A light copyedit for grammar and flow would be helpful to remove minor redundancies.
  - **Done for the entire manuscript**
- On generalizability, explicitly note that the sample (largely female, younger age group, single health system) may limit wider applicability, even though this is common in healthcare worker studies.
  - **Added at the end of the limitation section “The generalizability of these findings is limited by the sample characteristics, as participants were predominantly female, younger in age, and recruited from a single health system. While such demographic patterns are common in healthcare worker studies, they may restrict the applicability of results to broader or more diverse populations.”**
- Finally, the Conclusion could be strengthened by pointing more directly to how findings may inform workplace wellness policies, beyond the broad recommendation to support pleasurable activities.
  - **The last paragraph of the conclusion changed to: “In particular, our study demonstrates that *engaging in pleasurable activities* is associated with reduced declines in both physical health and psychological well-being. This finding could inform future workplace wellness policies. Organizations should consider implementing structured programs that encourage participation in enjoyable activities during the workday, such as group exercise sessions, creative workshops, or mindfulness breaks as part of wellness initiatives especially during times of increased stress.”**

With these minor revisions, I believe the manuscript will be ready for acceptance.

Best regards,

- - - - - - - - - - - - - - - -

Thank you and if you need further assistance, please let me know.

Kind regards, 

Nelson Leoja
Straive Editorial Assistant

PLOS ONE | plosone@plos.org
Empowering researchers to transform science

Case Number: 09185848

thread::gXBOYPpJcsaKWWU7vc2Caho::
