# Supplementary material for: Association of health behaviors with healthcare workers’ physical and psychological well-being: Learning from the COVID-19 pandemic
Source: PLoS One. 2025 Oct 31;20(10):e0334752. doi: 10.1371/journal.pone.0334752 (PMC12578231; doi:10.1371/journal.pone.0334752)
Supplement: Peer review history

# Editor report: Decision Letter 0

**DOI:** 10.1371/journal.pone.0334752.r002

**Authors:** Roy Rillera Marzo

11 Mar 2024

Dear Dr. Mulhem,

Thank you for submitting your manuscript to PLOS ONE. After careful consideration, we feel that it has merit but does not fully meet PLOS ONE’s publication criteria as it currently stands. Therefore, we invite you to submit a revised version of the manuscript that addresses the points raised during the review process.

Please submit your revised manuscript by Apr 25 2024 11:59PM. If you will need more time than this to complete your revisions, please reply to this message or contact the journal office at plosone@plos.org . When you're ready to submit your revision, log on to https://www.editorialmanager.com/pone/ and select the 'Submissions Needing Revision' folder to locate your manuscript file.

- A rebuttal letter that responds to each point raised by the academic editor and reviewer(s). You should upload this letter as a separate file labeled 'Response to Reviewers'.
- A marked-up copy of your manuscript that highlights changes made to the original version. You should upload this as a separate file labeled 'Revised Manuscript with Track Changes'.
- An unmarked version of your revised paper without tracked changes. You should upload this as a separate file labeled 'Manuscript'.

If applicable, we recommend that you deposit your laboratory protocols in protocols.io to enhance the reproducibility of your results. Protocols.io assigns your protocol its own identifier (DOI) so that it can be cited independently in the future. For instructions see: https://journals.plos.org/plosone/s/submission-guidelines#loc-laboratory-protocols . Additionally, PLOS ONE offers an option for publishing peer-reviewed Lab Protocol articles, which describe protocols hosted on protocols.io. Read more information on sharing protocols at https://plos.org/protocols?utm_medium=editorial-email&utm_source=authorletters&utm_campaign=protocols .

We look forward to receiving your revised manuscript.

Kind regards,

Roy Rillera Marzo, Ph.D.

Academic Editor

PLOS ONE

Journal Requirements:

When submitting your revision, we need you to address these additional requirements.

1. Please ensure that your manuscript meets PLOS ONE's style requirements, including those for file naming. The PLOS ONE style templates can be found at 

https://journals.plos.org/plosone/s/file?id=wjVg/PLOSOne_formatting_sample_main_body.pdf and 

https://journals.plos.org/plosone/s/file?id=ba62/PLOSOne_formatting_sample_title_authors_affiliations.pdf

2. Please include your full ethics statement in the ‘Methods’ section of your manuscript file. In your statement, please include the full name of the IRB or ethics committee who approved or waived your study, as well as whether or not you obtained informed written or verbal consent. If consent was waived for your study, please include this information in your statement as well. 

3. Please include captions for your Supporting Information files at the end of your manuscript, and update any in-text citations to match accordingly. Please see our Supporting Information guidelines for more information: http://journals.plos.org/plosone/s/supporting-information. 

4. Please review your reference list to ensure that it is complete and correct. If you have cited papers that have been retracted, please include the rationale for doing so in the manuscript text, or remove these references and replace them with relevant current references. Any changes to the reference list should be mentioned in the rebuttal letter that accompanies your revised manuscript. If you need to cite a retracted article, indicate the article’s retracted status in the References list and also include a citation and full reference for the retraction notice.

Additional Editor Comments:

There are areas where it could be improved for clarity and depth of your manuscript.

Introduction

1. The introduction broadly mentions the high vulnerability of nurses and midwives to mental health issues due to job characteristics and provides global prevalence rates. It would benefit from a more detailed exploration of the specific contextual factors within the Vietnamese healthcare system that may exacerbate these issues. Highlighting unique challenges faced by healthcare workers in Vietnam, such as specific cultural, economic, or healthcare policy factors, could provide a deeper understanding of the study's relevance and the need for targeted interventions in this particular setting.

2. While the introduction briefly mentions the impact of mental health issues on care quality and treatment outcomes, it lacks a detailed discussion on how anxiety, depression, and stress specifically affect the professional and personal lives of nurses and midwives. Expanding on the consequences, such as the potential for increased error rates, reduced patient satisfaction, burnout, and turnover, would strengthen the argument for the importance of addressing these issues.

3. The introduction could further emphasize what new contributions this study makes to the existing body of literature. While it mentions the study's aim to investigate depression, anxiety, and stress among healthcare workers in a specific hospital in Hanoi, Vietnam, it does not clearly state how this study fills gaps in the current research landscape. For example, detailing how this study's findings could inform specific policy changes or interventions in the Vietnamese context would highlight its significance and utility beyond adding to prevalence data.

Methods:

1. The use of a convenience sampling method is acknowledged, but the text lacks a discussion of how this choice may limit the generalizability of the study findings. An elaboration on the potential biases introduced by convenience sampling, such as selection bias and non-representativeness of the sample, would provide readers with a clearer understanding of the study's limitations and the extent to which the results can be generalized to the broader population of healthcare workers in Vietnam or similar contexts.

2. While the study mentions the formula used for estimating the sample size and the parameters considered (confidence level, expected percentage, and relative error level), it does not provide a detailed explanation or justification for choosing these specific parameters, especially the expected percentage of healthcare workers suffering from anxiety based on previous studies. Expanding on why these parameters were selected and how they align with the study's objectives could strengthen the rationale behind the sample size calculation and enhance the methodological transparency.

3. The section briefly mentions the use of the Tobit censored regression model to estimate linear relationships between variables, considering the censoring in the DASS-21 subscales. However, it lacks a comprehensive explanation of the rationale for choosing this specific statistical method over others and does not discuss the assumptions underlying the Tobit model. A more thorough discussion of the model's suitability for the data, including any assumptions made and how they were verified or addressed, would provide a stronger foundation for the statistical analysis and interpretation of the results.

Discussion

1. the discussion emphasizes the novelty of the study, it could benefit from a more thorough comparison with existing research, particularly studies conducted in similar healthcare contexts or developing countries. This comparative analysis could help identify unique factors affecting mental health in the Vietnamese healthcare setting and how these findings contribute to or diverge from global patterns in healthcare worker mental health. Please use ALL the 8 references below to have a comprehensive discussion:

A. Mental health, risk perception, and coping strategies among healthcare workers in Egypt during the COVID-19 pandemic. PLOS ONE. https://doi.org/10.1371/journal.pone.0282264

B. Demographic and work-related factors associated with burnout, resilience, and quality of life among healthcare workers during the COVID-19 pandemic: A cross sectional study from Malaysia. Sec. Public Health Education and Promotion. https://doi.org/10.3389/fpubh.2022.1021495

C. Burnout, resilience and the quality of life among Malaysian healthcare workers during the COVID-19 pandemic. Frontiers in Public Health, 30 November 2022. Sec. Public Health Education and Promotion. https://doi.org/10.3389/fpubh.2022.1021497

D. Palestinian Healthcare Workers Mental Health Challenges during COVID-19 Pandemic: A Cross-Sectional Study. Medical Research Archives. October 31, 2022. https://esmed.org/MRA/index.php/mra/article/view/3125

E. Risk perception, mental health impacts and coping strategies during COVID-19 pandemic among Filipino healthcare workers. https://journals.sagepub.com/doi/full/10.4081/jphr.2021.2604

F. Impact of COVID-19 pandemic on mental health, risk perception and coping strategies among health care workers in Albania - evidence that needs attention. Clinical Epidemiology and Global Health Volume 12, October–December 2021, 100824. Doi: https://doi.org/10.1016/j.cegh.2021.100824

G. How healthcare workers are coping with mental health challenges during COVID-19 pandemic? - A cross-sectional multi-countries study.. 2021 May 7:100759. doi: https://doi.org/10.1016/j.cegh.2021.100759

H. The immediate impact of COVID-19 on mental health and its associated factors among healthcare workers: A global perspective across 31 countries.. Journal of Global Health. August 23, 2021. https://www.ncbi.nlm.nih.gov/pmc/articles/PMC7649521/

2. The discussion mentions associated factors like age, type of contract, years of working, and health issues but does not delve deeply into the causal mechanisms or underlying reasons that might explain these associations. A more detailed exploration of why these factors impact mental health in the specified context would provide a richer understanding and inform more targeted interventions.

Conclusion

1. The conclusion would benefit from addressing the broader implications of the findings for healthcare policy and workforce management. Highlighting specific steps for intervention and the potential for scalability in similar settings would enhance its applicability and impact.

[Note: HTML markup is below. Please do not edit.]

[NOTE: If reviewer comments were submitted as an attachment file, they will be attached to this email and accessible via the submission site. Please log into your account, locate the manuscript record, and check for the action link "View Attachments". If this link does not appear, there are no attachment files.]

While revising your submission, please upload your figure files to the Preflight Analysis and Conversion Engine (PACE) digital diagnostic tool, https://pacev2.apexcovantage.com/ . PACE helps ensure that figures meet PLOS requirements. To use PACE, you must first register as a user. Registration is free. Then, login and navigate to the UPLOAD tab, where you will find detailed instructions on how to use the tool. If you encounter any issues or have any questions when using PACE, please email PLOS at figures@plos.org

# Author comment: Author response to Decision Letter 1

**DOI:** 10.1371/journal.pone.0334752.r003

24 May 2024

we received editorial comments for a different manuscript. the PLOS One senior editor asked that we resubmit the manuscript.

# Peer review: Decision Letter 1

**DOI:** 10.1371/journal.pone.0334752.r004

**Authors:** Anna Rachel Conolly

9 Oct 2024

Dear Dr. Mulhem,

Thank you for submitting your manuscript to PLOS ONE. After careful consideration, we feel that it has merit but does not fully meet PLOS ONE’s publication criteria as it currently stands. Therefore, we invite you to submit a revised version of the manuscript that addresses the points raised during the review process.

Please submit your revised manuscript by Nov 23 2024 11:59PM. If you will need more time than this to complete your revisions, please reply to this message or contact the journal office at plosone@plos.org . When you're ready to submit your revision, log on to https://www.editorialmanager.com/pone/ and select the 'Submissions Needing Revision' folder to locate your manuscript file.

- A rebuttal letter that responds to each point raised by the academic editor and reviewer(s). You should upload this letter as a separate file labeled 'Response to Reviewers'.
- A marked-up copy of your manuscript that highlights changes made to the original version. You should upload this as a separate file labeled 'Revised Manuscript with Track Changes'.
- An unmarked version of your revised paper without tracked changes. You should upload this as a separate file labeled 'Manuscript'.

If you would like to make changes to your financial disclosure, please include your updated statement in your cover letter. Guidelines for resubmitting your figure files are available below the reviewer comments at the end of this letter.

If applicable, we recommend that you deposit your laboratory protocols in protocols.io to enhance the reproducibility of your results. Protocols.io assigns your protocol its own identifier (DOI) so that it can be cited independently in the future. For instructions see: https://journals.plos.org/plosone/s/submission-guidelines#loc-laboratory-protocols . Additionally, PLOS ONE offers an option for publishing peer-reviewed Lab Protocol articles, which describe protocols hosted on protocols.io. Read more information on sharing protocols at https://plos.org/protocols?utm_medium=editorial-email&utm_source=authorletters&utm_campaign=protocols .

We look forward to receiving your revised manuscript.

Kind regards,

Anna Rachel Conolly, PhD, MSc, PG Dip, BA (hons)

Academic Editor

PLOS ONE

**Additional Editor Comments:**

Dear Authors,

Thank you for giving me the chance to review your paper which highlights important findings about the importance of positive health behaviours, offering valuable insights into potential protective factors during crises. Your study is timely, addressing the significant toll that the pandemic has had on healthcare workers, who were particularly vulnerable to stress, burnout, and health issues due to their frontline roles. By focusing on how they coped with stress using health behaviours, the study offers actionable insights for improving well-being during crises. I suggest major revisions to your paper in-line with the the comments the first author has made.

Once the revisions suggested by reviewer one have been actioned I believe the paper will make a valuable contribution to understanding how healthcare workers' health behaviours influenced their psychological and physical health during the pandemic.

Best wishes

Anna Conolly

[Note: HTML markup is below. Please do not edit.]

Reviewers' comments:

Reviewer's Responses to Questions

**Comments to the Author**

Reviewer #1: (No Response)

**********

2. Is the manuscript technically sound, and do the data support the conclusions?

Reviewer #1: No

**********

3. Has the statistical analysis been performed appropriately and rigorously?

Reviewer #1: No

**********

4. Have the authors made all data underlying the findings in their manuscript fully available?

The PLOS Data policy

Reviewer #1: No

**********

5. Is the manuscript presented in an intelligible fashion and written in standard English?

Reviewer #1: Yes

**********

**Reviewer #1:**  I am grateful for the chance to contribute to the peer-review process of this manuscript.

The general aim of the work is quite clear: to see if there is an association between health behaviors during the pandemic (or rather, the second wave of the pandemic) and self-reported physical and psychological health.

However, there is a certain ambiguity in the objectives, given that analyzes referring to during the pandemic alternate with analyzes in which changes from before to during the pandemic are evaluated. I suggest clarifying clearly in the Introduction what scientific questions the authors aim to answer.

Furthermore, many details of the study and data analysis are omitted and it is therefore difficult to understand the significance of the results.

Plese, see more detailed remarks and suggestions in the attached file.

**********

what does this mean? ). If published, this will include your full peer review and any attached files.

If you choose “no”, your identity will remain anonymous but your review may still be made public.

**Do you want your identity to be public for this peer review?** For information about this choice, including consent withdrawal, please see our Privacy Policy

Reviewer #1: No

**********

[NOTE: If reviewer comments were submitted as an attachment file, they will be attached to this email and accessible via the submission site. Please log into your account, locate the manuscript record, and check for the action link "View Attachments". If this link does not appear, there are no attachment files.]

While revising your submission, please upload your figure files to the Preflight Analysis and Conversion Engine (PACE) digital diagnostic tool, https://pacev2.apexcovantage.com/ . PACE helps ensure that figures meet PLOS requirements. To use PACE, you must first register as a user. Registration is free. Then, login and navigate to the UPLOAD tab, where you will find detailed instructions on how to use the tool. If you encounter any issues or have any questions when using PACE, please email PLOS at figures@plos.org . Please note that Supporting Information files do not need this step.

Attachment Submitted filename: PONE-D-24-07740-Review.pdf

# Author comment: Author response to Decision Letter 2

**DOI:** 10.1371/journal.pone.0334752.r005

24 Mar 2025

Thank you to Reviewer 1 for detailed feedback and concise suggestions.

we made all changes requested including new data analysis.

Attachment Submitted filename: PLOS ONE response to reviewer 3 11 25.docx

# Peer review: Decision Letter 2

**DOI:** 10.1371/journal.pone.0334752.r006

**Authors:** Zypher Jude G. Regencia

22 Apr 2025

Dear Dr. Mulhem,

Thank you for submitting your manuscript to PLOS ONE. After careful consideration, we feel that it has merit but does not fully meet PLOS ONE’s publication criteria as it currently stands. Therefore, we invite you to submit a revised version of the manuscript that addresses the points raised during the review process.

Please submit your revised manuscript by Jun 06 2025 11:59PM. If you will need more time than this to complete your revisions, please reply to this message or contact the journal office at plosone@plos.org . When you're ready to submit your revision, log on to https://www.editorialmanager.com/pone/ and select the 'Submissions Needing Revision' folder to locate your manuscript file.

- A rebuttal letter that responds to each point raised by the academic editor and reviewer(s). You should upload this letter as a separate file labeled 'Response to Reviewers'.
- A marked-up copy of your manuscript that highlights changes made to the original version. You should upload this as a separate file labeled 'Revised Manuscript with Track Changes'.
- An unmarked version of your revised paper without tracked changes. You should upload this as a separate file labeled 'Manuscript'.

If applicable, we recommend that you deposit your laboratory protocols in protocols.io to enhance the reproducibility of your results. Protocols.io assigns your protocol its own identifier (DOI) so that it can be cited independently in the future. For instructions see: https://journals.plos.org/plosone/s/submission-guidelines#loc-laboratory-protocols . Additionally, PLOS ONE offers an option for publishing peer-reviewed Lab Protocol articles, which describe protocols hosted on protocols.io. Read more information on sharing protocols at https://plos.org/protocols?utm_medium=editorial-email&utm_source=authorletters&utm_campaign=protocols .

We look forward to receiving your revised manuscript.

Kind regards,

Zypher Jude G. Regencia, Ph.D.

Academic Editor

PLOS ONE

Journal Requirements:

Please review your reference list to ensure that it is complete and correct. If you have cited papers that have been retracted, please include the rationale for doing so in the manuscript text, or remove these references and replace them with relevant current references. Any changes to the reference list should be mentioned in the rebuttal letter that accompanies your revised manuscript. If you need to cite a retracted article, indicate the article’s retracted status in the References list and also include a citation and full reference for the retraction notice.

Reviewers' comments:

Reviewer's Responses to Questions

**Comments to the Author**

Reviewer #1: (No Response)

Reviewer #2: (No Response)

**********

2. Is the manuscript technically sound, and do the data support the conclusions?

Reviewer #1: Yes

Reviewer #2: Yes

**********

3. Has the statistical analysis been performed appropriately and rigorously?

Reviewer #1: Yes

Reviewer #2: Yes

**********

4. Have the authors made all data underlying the findings in their manuscript fully available?

The PLOS Data policy

Reviewer #1: Yes

Reviewer #2: No

**********

5. Is the manuscript presented in an intelligible fashion and written in standard English?

Reviewer #1: Yes

Reviewer #2: Yes

**********

Reviewer #1: First of all, I would like to thank the authors for their extensive review and detailed responses to my comments. I have just a few small comments to make, to conclude.

1. Section "Statistical analysis": Please, move the sentence "Residual versus fits plots and quantile

quantile plots for all linear models were inspected to verify the model assumptions were reasonable. All

analyses were performed using R Statistical Software (v4.2.2; R Core Team 2022)." to the subsession "Association Between Change in Physical Health and Psychological Well-Being and Health Behaviors", where linear models are introduced

2. Table 1. Please, note that the Fisher test for tables larger than 2x2 is called the Fisher-Freeman-Halton test (Freeman G.H. and Halton J.H. (1951), Note on an exact treatment of contingency, goodness of fit and other problems of significance. Biometrika 38:141-149).

3. The opening sentence of the discussion: This study found that healthcare workers who regularly engaged in positive health behaviors and coping strategies during COVID-19 did better psychologically and physically **both** before and during COVID-19" is not supported by the data. In fact, engagin in health behaviors and coping strategies during COVID-19 was associated to CHANGES in physical health adn psychologicalwell-being. Please, refrase this sentence.

4. Figure 6: I suggest completing the caption "Engaged ...." by adding **during the pandemic**. I also suggest moving it and the "No/Yes" legend **above** each of the three graphs. In its current position, the caption seems to suggest that engagement was detected both before and during the pandemic. Below each graph, simply indicate "self-reported physical health" or something like this.

Reviewer #2: 1. The manuscript reads much better now—thank you for addressing the previous round of comments. The flow of the introduction and the rationale is clearer.

2. I appreciate the effort in the new data analyses. Including adjusted linear models for each health behavior is a solid addition and strengthens the statistical rigor.

3. The distinction between physical and psychological well-being outcomes is clearly articulated, although some transitions between these concepts in the discussion could still be tightened.

4. I still feel the lack of baseline data on health behaviors (i.e., pre-pandemic) is a notable limitation—perhaps this could be more explicitly acknowledged upfront in the limitations section, not just at the end.

5. The finding that only pleasurable activities had a protective effect on psychological well-being is intriguing. It might be worth briefly discussing why other commonly cited interventions (e.g., sleep, exercise) didn’t have similar effects here.

6. The explanation about why mindfulness/relaxation exercises may not have shown significant results is well-reasoned. Good job offering a plausible rationale based on lack of formal training.

7. There’s a lot of useful data in the figures, but some are dense. Simplifying captions and highlighting the key takeaway from each figure would help guide the reader better.

8. The manuscript could benefit from a light copyedit for grammar and flow. Some sections still contain redundant phrases or awkward syntax (e.g., “did reported better”).

9. On generalizability—can you comment on how representative this sample is of U.S. healthcare workers? The demographics lean heavily female and young, which is common but may still limit wider applicability.

10. The conclusion is appropriately cautious, though I’d suggest briefly noting how these findings could inform specific workplace wellness policies moving forward, beyond general suggestions.

**********

what does this mean? ). If published, this will include your full peer review and any attached files.

If you choose “no”, your identity will remain anonymous but your review may still be made public.

**Do you want your identity to be public for this peer review?** For information about this choice, including consent withdrawal, please see our Privacy Policy

Reviewer #1: **Yes: ** Antonella Bodini

Reviewer #2: No

**********

While revising your submission, please upload your figure files to the Preflight Analysis and Conversion Engine (PACE) digital diagnostic tool, https://pacev2.apexcovantage.com/ . PACE helps ensure that figures meet PLOS requirements. To use PACE, you must first register as a user. Registration is free. Then, login and navigate to the UPLOAD tab, where you will find detailed instructions on how to use the tool. If you encounter any issues or have any questions when using PACE, please email PLOS at figures@plos.org

# Author comment: Author response to Decision Letter 3

**DOI:** 10.1371/journal.pone.0334752.r007

30 Apr 2025

Reviewer #1: First of all, I would like to thank the authors for their extensive review and detailed responses to my comments. I have just a few small comments to make, to conclude.

Thank you

1. Section "Statistical analysis": Please, move the sentence "Residual versus fits plots and quantile

quantile plots for all linear models were inspected to verify the model assumptions were reasonable. All analyses were performed using R Statistical Software (v4.2.2; R Core Team 2022)." to the subsession "Association Between Change in Physical Health and Psychological Well-Being and Health Behaviors", where linear models are introduced

Sentence moved as recommended

2. Table 1. Please, note that the Fisher test for tables larger than 2x2 is called the Fisher-Freeman-Halton test (Freeman G.H. and Halton J.H. (1951), Note on an exact treatment of contingency, goodness of fit and other problems of significance. Biometrika 38:141-149).

Changed Fisher exact to Fisher-Freeman-Halton

3. The opening sentence of the discussion: This study found that healthcare workers who regularly engaged in positive health behaviors and coping strategies during COVID-19 did better psychologically and physically **both** before and during COVID-19" is not supported by the data. In fact, engagin in health behaviors and coping strategies during COVID-19 was associated to CHANGES in physical health adn psychologicalwell-being. Please, refrase this sentence.

Sentence changed to “engaging in positive health behaviors and coping strategies during COVID-19 had a positive impact on physical and psychological well-being”

4. Figure 6: I suggest completing the caption "Engaged ...." by adding **during the pandemic**. I also suggest moving it and the "No/Yes" legend **above** each of the three graphs. In its current position, the caption seems to suggest that engagement was detected both before and during the pandemic. Below each graph, simply indicate "self-reported physical health" or something like this.

Added “during the pandemic” to the caption.

We have yes/no at the bottom of each graph.

Changed “physical health” to “self-reported physical health” in the caption.

Reviewer #2: 1. The manuscript reads much better now—thank you for addressing the previous round of comments. The flow of the introduction and the rationale is clearer.

Thank you

2. I appreciate the effort in the new data analyses. Including adjusted linear models for each health behavior is a solid addition and strengthens the statistical rigor.

Thank you

3. The distinction between physical and psychological well-being outcomes is clearly articulated, although some transitions between these concepts in the discussion could still be tightened.

Please provide specific areas in the discussion where this can be done

4. I still feel the lack of baseline data on health behaviors (i.e., pre-pandemic) is a notable limitation—perhaps this could be more explicitly acknowledged upfront in the limitations section, not just at the end.

This issue is discussed clearly in the limitation section, and it occupies most of the section, we don’t feel that moving it up couple lines will make any significant difference.

5. The finding that only pleasurable activities had a protective effect on psychological well-being is intriguing. It might be worth briefly discussing why other commonly cited interventions (e.g., sleep, exercise) didn’t have similar effects here.

We already covered this point in the discussion “It is unclear why these health behaviors appear to counteract the decline in physical health during the pandemic but not psychological well-being. A possible explanation is that there is a two-way relationship between health behaviors and health status. People who were feeling physically well may have been better positioned to engage in an array of healthy behaviors and coping strategies whereas the same may not have been true for those who were psychologically doing well.”

6. The explanation about why mindfulness/relaxation exercises may not have shown significant results is well-reasoned. Good job offering a plausible rationale based on lack of formal training.

Thank you

7. There’s a lot of useful data in the figures, but some are dense. Simplifying captions and highlighting the key takeaway from each figure would help guide the reader better.

We made changes to captions as recommended by reviewer 1. Please be more specific about changes you recommend.

8. The manuscript could benefit from a light copyedit for grammar and flow. Some sections still contain redundant phrases or awkward syntax (e.g., “did reported better”).

We reviewed the manuscript and did not find “did reported better” phrase.

9. On generalizability—can you comment on how representative this sample is of U.S. healthcare workers? The demographics lean heavily female and young, which is common but may still limit wider applicability.

The sample collected from one large health system in Southeast Michigan, which is a factor in limiting generalizability.

10. The conclusion is appropriately cautious, though I’d suggest briefly noting how these findings could inform specific workplace wellness policies moving forward, beyond general suggestions.

Changed the last sentence to: “This finding could inform specific workplace wellness policies moving forward. Interventions should focus on creating mechanisms to increase opportunities for engaging in pleasurable activities, both in the workplace and at home, especially during times of increased stress.”

Attachment Submitted filename: Reviewers comment 2.docx

# Peer review: Decision Letter 3

**DOI:** 10.1371/journal.pone.0334752.r008

**Authors:** Zypher Jude G. Regencia

10 Aug 2025

Dear Dr. Mulhem,

Thank you for submitting your manuscript to PLOS ONE. After careful consideration, we feel that it has merit but does not fully meet PLOS ONE’s publication criteria as it currently stands. Therefore, we invite you to submit a revised version of the manuscript that addresses the points raised during the review process.

Please submit your revised manuscript by Sep 24 2025 11:59PM. If you will need more time than this to complete your revisions, please reply to this message or contact the journal office at plosone@plos.org . When you're ready to submit your revision, log on to https://www.editorialmanager.com/pone/ and select the 'Submissions Needing Revision' folder to locate your manuscript file.

- A rebuttal letter that responds to each point raised by the academic editor and reviewer(s). You should upload this letter as a separate file labeled 'Response to Reviewers'.
- A marked-up copy of your manuscript that highlights changes made to the original version. You should upload this as a separate file labeled 'Revised Manuscript with Track Changes'.
- An unmarked version of your revised paper without tracked changes. You should upload this as a separate file labeled 'Manuscript'.

If applicable, we recommend that you deposit your laboratory protocols in protocols.io to enhance the reproducibility of your results. Protocols.io assigns your protocol its own identifier (DOI) so that it can be cited independently in the future. For instructions see: https://journals.plos.org/plosone/s/submission-guidelines#loc-laboratory-protocols . Additionally, PLOS ONE offers an option for publishing peer-reviewed Lab Protocol articles, which describe protocols hosted on protocols.io. Read more information on sharing protocols at https://plos.org/protocols?utm_medium=editorial-email&utm_source=authorletters&utm_campaign=protocols .

We look forward to receiving your revised manuscript.

Kind regards,

Zypher Jude G. Regencia, Ph.D.

Academic Editor

PLOS ONE

Journal Requirements:

1. If the reviewer comments include a recommendation to cite specific previously published works, please review and evaluate these publications to determine whether they are relevant and should be cited. There is no requirement to cite these works unless the editor has indicated otherwise. 

2. Please review your reference list to ensure that it is complete and correct. If you have cited papers that have been retracted, please include the rationale for doing so in the manuscript text, or remove these references and replace them with relevant current references. Any changes to the reference list should be mentioned in the rebuttal letter that accompanies your revised manuscript. If you need to cite a retracted article, indicate the article’s retracted status in the References list and also include a citation and full reference for the retraction notice.

Reviewers' comments:

Reviewer's Responses to Questions

**Comments to the Author**

Reviewer #1: All comments have been addressed

Reviewer #2: (No Response)

**********

2. Is the manuscript technically sound, and do the data support the conclusions?

Reviewer #1: (No Response)

Reviewer #2: Yes

**********

3. Has the statistical analysis been performed appropriately and rigorously?

Reviewer #1: (No Response)

Reviewer #2: Yes

**********

4. Have the authors made all data underlying the findings in their manuscript fully available?

The PLOS Data policy

Reviewer #1: (No Response)

Reviewer #2: No

**********

5. Is the manuscript presented in an intelligible fashion and written in standard English?

Reviewer #1: (No Response)

Reviewer #2: Yes

**********

Reviewer #1: (No Response)

Reviewer #2: Overall, this is a well-prepared and respectful response to the reviewers' comments. The authors made a genuine effort to address each point in detail, and their tone throughout is measured, appreciative, and constructive. That said, a few elements could still be improved for clarity and completeness.

What was done well:

The responses were comprehensive and non-defensive. You took the time to clarify your rationale where needed and accepted suggestions gracefully, especially in response to Reviewer #2’s more critical points.

Your justification for maintaining the 14-day data collection window was clear and appropriate. Linking it to the academic calendar helped contextualize your constraints without sounding dismissive.

The explanation for using partial correlations was concise but sufficient, especially with the supporting reference you added. It showed that your decision was methodologically grounded.

You incorporated limitations raised by the reviewers into the manuscript itself, including the issues on generalizability and study duration, which reflects scientific transparency.

The addition of references regarding perceived stress variability across the academic term was thoughtful and shows that you are engaged with the literature and the context of your sample.

Points to consider revising or strengthening further:

In your response about the representativeness of the sample, you stated confidence in its generalizability. This could benefit from a more cautious tone, as convenience sampling always introduces potential bias. Acknowledging this more explicitly would strengthen your argument.

Reviewer #1 raised a concern regarding the theoretical grounding for the mediator sequence. Your response here felt brief. Consider adding more depth by citing theoretical or empirical literature that supports the assumed temporal ordering, even if it’s based on plausibility rather than longitudinal evidence.

Where you mention having updated the abstract and results, it might be helpful to specify exactly what was revised. This level of detail gives reviewers reassurance that their feedback was not only noted but actively integrated into the manuscript.

While your justification for the cross-sectional design is reasonable, it would be stronger if you included a brief statement on how future research could build on this, e.g., through longitudinal follow-up.

**********

what does this mean? ). If published, this will include your full peer review and any attached files.

If you choose “no”, your identity will remain anonymous but your review may still be made public.

**Do you want your identity to be public for this peer review?** For information about this choice, including consent withdrawal, please see our Privacy Policy

Reviewer #1: **Yes: ** Antonella Bodini

Reviewer #2: No

**********

[NOTE: If reviewer comments were submitted as an attachment file, they will be attached to this email and accessible via the submission site. Please log into your account, locate the manuscript record, and check for the action link "View Attachments". If this link does not appear, there are no attachment files.]

While revising your submission, please upload your figure files to the Preflight Analysis and Conversion Engine (PACE) digital diagnostic tool, https://pacev2.apexcovantage.com/ . PACE helps ensure that figures meet PLOS requirements. To use PACE, you must first register as a user. Registration is free. Then, login and navigate to the UPLOAD tab, where you will find detailed instructions on how to use the tool. If you encounter any issues or have any questions when using PACE, please email PLOS at figures@plos.org

# Author comment: Author response to Decision Letter 4

**DOI:** 10.1371/journal.pone.0334752.r009

16 Sep 2025

Dear Dr. Plos One team,

Thank you again for your careful revisions and responses. I apologize for the earlier oversight — I am currently reviewing many submissions, and I appreciate your patience. After reviewing the manuscript and the latest set of comments, I believe the paper is very close to acceptance and only requires minor revisions to further improve clarity and flow.

Thank you for your comments, we addressed your recommendations in bold below:

Please see the remaining points below:

• Move the sentence about residual versus fits plots, quantile–quantile plots, and the use of R software into the subsection where linear models are introduced.

o Sentence moved as requested

• For Table 1, update the terminology to refer to the Fisher–Freeman–Halton test when contingency tables are larger than 2×2.

o Added

• In the Discussion, the opening sentence suggests outcomes were tracked both before and during COVID. Since the data focus on change during the pandemic, please rephrase to avoid overstating.

o The opening sentence changed to: Consistent with previous studies from our group and others, healthcare workers reported high levels of work- related stress, low quality of their personal life, and low levels of psychological well-being and physical health during the COVID-19 pandemic (3, 4, 11, 12, 14).

• For Figure 6, add the phrase “during the pandemic” in the caption, shift the Yes/No legend above each graph, and clarify the x-axis label as “self-reported physical health.”

o Changes made to the figure and capture as suggested.

• The transitions between psychological well-being and physical health in the Discussion could be tightened to improve readability.

o We changed the transition sentence to: In contrast to psychological well-being, our assessment of factors affecting physical health revealed that……

• The limitation regarding the lack of baseline (pre-pandemic) health behavior data should be emphasized more prominently.

o We made minor edits, and we feel that this limitation is thoroughly addressed in the limitation section, that discussion constitutes more than two-thirds of that section.

• Since only pleasurable activities showed a protective effect on psychological well-being, a brief reflection on why other health behaviors (e.g., exercise, sleep) did not have similar effects would strengthen the Discussion.

o Added to the discussion “Other positive health behaviors (e.g., exercise, sleep) did not show a protective effect on psychological well-being, which may be explained by the fact that pleasurable activities provide more immediate or direct emotional relief from stress, whereas the benefits of exercise and sleep are often more gradual or less noticeable during periods of acute, ongoing stress.”

• The rationale regarding mindfulness/relaxation is clear and well-reasoned; just ensure it remains concise.

o Thank you

• Some figure captions could be simplified and should highlight the main takeaway for each figure.

o Changes made to simplify figures captions

• A light copyedit for grammar and flow would be helpful to remove minor redundancies.

o Done for the entire manuscript

• On generalizability, explicitly note that the sample (largely female, younger age group, single health system) may limit wider applicability, even though this is common in healthcare worker studies.

o Added at the end of the limitation section “The generalizability of these findings is limited by the sample characteristics, as participants were predominantly female, younger in age, and recruited from a single health system. While such demographic patterns are common in healthcare worker studies, they may restrict the applicability of results to broader or more diverse populations.”

• Finally, the Conclusion could be strengthened by pointing more directly to how findings may inform workplace wellness policies, beyond the broad recommendation to support pleasurable activities.

o The last paragraph of the conclusion changed to: “In particular, our study demonstrates that engaging in pleasurable activities is associated with reduced declines in both physical health and psychological well-being. This finding could inform future workplace wellness policies. Organizations should consider implementing structured programs that encourage participation in enjoyable activities during the workday, such as group exercise sessions, creative workshops, or mindfulness breaks as part of wellness initiatives especially during times of increased stress.”

With these minor revisions, I believe the manuscript will be ready for acceptance.

Best regards,

- - - - - - - - - - - - - - - -

Thank you and if you need further assistance, please let me know.

Kind regards,

Nelson Leoja

Straive Editorial Assistant

PLOS ONE | plosone@plos.org

Empowering researchers to transform science

Case Number: 09185848

thread::gXBOYPpJcsaKWWU7vc2Caho::

Attachment Submitted filename: PLOS ONE editors comments- reply.docx

# Editor report: Decision Letter 4

**DOI:** 10.1371/journal.pone.0334752.r010

**Authors:** Zypher Jude G. Regencia

1 Oct 2025

Association of Health Behaviors with Healthcare Workers’ Physical and Psychological Well-being: Learning from the COVID-19 Pandemic

PONE-D-24-07740R4

Dear Dr. Mulhem,

We’re pleased to inform you that your manuscript has been judged scientifically suitable for publication and will be formally accepted for publication once it meets all outstanding technical requirements.

Within one week, you’ll receive an e-mail detailing the required amendments. When these have been addressed, you’ll receive a formal acceptance letter and your manuscript will be scheduled for publication.

An invoice will be generated when your article is formally accepted. Please note, if your institution has a publishing partnership with PLOS and your article meets the relevant criteria, all or part of your publication costs will be covered. Please make sure your user information is up-to-date by logging into Editorial Manager at Editorial Manager®  and clicking the ‘Update My Information' link at the top of the page. For questions related to billing, please contact billing support .

If your institution or institutions have a press office, please notify them about your upcoming paper to help maximize its impact. If they’ll be preparing press materials, please inform our press team as soon as possible -- no later than 48 hours after receiving the formal acceptance. Your manuscript will remain under strict press embargo until 2 pm Eastern Time on the date of publication. For more information, please contact onepress@plos.org.

Kind regards,

Zypher Jude G. Regencia, Ph.D.

Academic Editor

PLOS ONE

Additional Editor Comments (optional):

Reviewers' comments:

# Editor report: Acceptance letter

**DOI:** 10.1371/journal.pone.0334752.r011

**Authors:** Zypher Jude G. Regencia

PONE-D-24-07740R4

PLOS ONE

Dear Dr. Mulhem,

I'm pleased to inform you that your manuscript has been deemed suitable for publication in PLOS ONE. Congratulations! Your manuscript is now being handed over to our production team.

At this stage, our production department will prepare your paper for publication. This includes ensuring the following:

* All references, tables, and figures are properly cited

* All relevant supporting information is included in the manuscript submission,

* There are no issues that prevent the paper from being properly typeset

You will receive further instructions from the production team, including instructions on how to review your proof when it is ready. Please keep in mind that we are working through a large volume of accepted articles, so please give us a few days to review your paper and let you know the next and final steps.

Lastly, if your institution or institutions have a press office, please let them know about your upcoming paper now to help maximize its impact. If they'll be preparing press materials, please inform our press team within the next 48 hours. Your manuscript will remain under strict press embargo until 2 pm Eastern Time on the date of publication. For more information, please contact onepress@plos.org.

You will receive an invoice from PLOS for your publication fee after your manuscript has reached the completed accept phase. If you receive an email requesting payment before acceptance or for any other service, this may be a phishing scheme. Learn how to identify phishing emails and protect your accounts at https://explore.plos.org/phishing.

If we can help with anything else, please email us at customercare@plos.org.

Thank you for submitting your work to PLOS ONE and supporting open access.

Kind regards,

PLOS ONE Editorial Office Staff

on behalf of

Dr. Zypher Jude G. Regencia

Academic Editor

PLOS ONE
